# *S*-1-Propenylcysteine promotes IL-10-induced M2c macrophage polarization through prolonged activation of IL-10R/STAT3 signaling

**DOI:** 10.1038/s41598-021-01866-3

**Published:** 2021-11-17

**Authors:** Satomi Miki, Jun-ichiro Suzuki, Miyuki Takashima, Mari Ishida, Hiroki Kokubo, Masao Yoshizumi

**Affiliations:** 1 Department of Cardiovascular Physiology and Medicine, Graduate School of Biomedical and Health Sciences, Hiroshima University, 1-2-3 Kasumi, Minami-ku, Hiroshima-shi, Hiroshima, 734-8551 Japan; 2 Central Research Institute, Wakunaga Pharmaceutical Co., Ltd., 1624 Shimokotachi, Koda-cho, Akitakata-shi, Hiroshima, 739-1195 Japan

**Keywords:** Immunology, Cardiology

## Abstract

Atherosclerosis is a chronic inflammatory disease that may lead to the development of serious cardiovascular diseases. Aged garlic extract (AGE) has been reported to ameliorate atherosclerosis, although its mode of action remains unclear. We found that AGE increased the mRNA or protein levels of arginase1 (Arg1), interleukin-10 (IL-10), CD206 and hypoxia-inducible factor 2α (HIF2α) and decreased that of CD68, HIF1α and inducible nitric oxide synthase in the aorta and spleen of apolipoprotein E knockout mice. We also found that *S*-1-propenylcysteine (S1PC), a characteristic sulfur compound in AGE, increased the level of IL-10-induced *Arg1* mRNA and the extent of M2c-like macrophage polarization in vitro. In addition, S1PC increased the population of M2c-like macrophages, resulting in suppressed the population of M1-like macrophages and decreased lipopolysaccharide-induced production of pro-inflammatory cytokines. These effects were accompanied by prolonged phosphorylation of the IL-10 receptor α (IL-10Rα) and signal transducer and activator of transcription 3 (STAT3) that inhibited the interaction between IL-10Rα and Src homology-2-containing inositol 5’-phosphatase 1 (SHIP1). In addition, administration of S1PC elevated the M2c/M1 macrophage ratio in senescence-accelerated mice. These findings suggest that S1PC may help improve atherosclerosis due to its anti-inflammatory effect to promote IL-10-induced M2c macrophage polarization.

## Introduction

Atherosclerosis is a chronic inflammatory disease that may lead to the development of life-threatening illnesses such as coronary artery disease (CAD), peripheral artery disease (PAD), and cerebrovascular disease^1,2^. In the early stages of atherosclerosis, oxidized low density lipoproteins (oxLDL) induce inflammation in arterial walls, causing augmented infiltration of monocytes into the sub-endothelial space and differentiation of monocytes into macrophages. Subsequently, the arterial macrophages are transformed into foam cells, a process that is accompanied by the upregulation of several genes, such as CD36 and CD68^2–6^. The foam cells then secrete pro-inflammatory cytokines, including interleukin (IL)-1β, IL-6, and tumor necrosis factor-α (TNF-α), into the arterial intima, promoting the formation of atherosclerotic plaques^1,7^. The resulting chronic inflammation promotes the release of damage-associated molecular patterns (DAMPs), such as nucleic acids and proteins from dead cells, as well as senescence-associated secretory phenotype (SASP) factors, such as inflammatory cytokines, growth factors, and proteases from senescent cells. These accelerate vascular aging by creating a positive feedback loop and eventually exacerbating plaque formation^8,9^.

Macrophages can be polarized to two major phenotypes depending on the microenvironmental conditions: pro-inflammatory M1 and anti-inflammatory M2 macrophages^10–12^. It has been reported that the development of atherosclerosis alters the ratio of polarized macrophages^13,14^. M1 macrophages promote the formation of atherosclerotic plaques by sustaining inflammation, whereas M2 macrophages aid the regression of atherosclerotic plaques by promoting tissue repair, anti-inflammatory cytokine release, and efferocytosis through phagocytosis of apoptotic cells^4,15–17^. Specifically, M2c macrophages inhibit the accumulation of foam cells by inducing mer tyrosine kinase (MerTK)-dependent efferocytosis^18,19^. The polarization of macrophages to pro-inflammatory M1 macrophages expressing TNF-α, CD68, IL-18, hypoxia-inducible factor 1α (HIF1α), C–C motif chemokine ligand 5 (CCL5), CD86, and nitric oxide (NO) is elicited by exposure to pro-inflammatory cytokines such as interferon-γ (IFN-γ) and TNF-α^11,12,20–24^. On the other hand, anti-inflammatory cytokine IL-10 induces polarization to anti-inflammatory M2c macrophages that produce IL-10, transforming growth factor-β (TGF-β), arginase-1 (Arg1), CD206, HIF2α, scavenger receptor AI (SR-AI), Suppressor of cytokine signaling 3 (SOCS3), and CD150^11,12,22,24–27^.

The IL-10 receptor (IL-10R) consists of a tetrameric complex with two ligand-binding subunits (IL-10Rα) and two accessory signaling subunits (IL-10Rβ)^28–31^. The binding of IL-10 to the extracellular domain of IL-10Rα results in the phosphorylation of janus kinase-1 (JAK1) and tyrosine kinase-2 (TYK2), which interact with IL-10Rα and IL-10Rβ, respectively^29–31^. Activated JAK1 then phosphorylates tyrosine residues such as Tyr^446^ and Tyr^496^ in the intracellular domain of the IL-10Rα subunits, leading to interactions between transcription factor signal transducer and activator of transcription 3 (STAT3) via the Src homology-2 (SH2) domain^31–33^. Additionally, phosphorylation of STAT3 at Tyr^705^ by JAK1 causes STAT3 dimerization in the cytoplasm. The dimerized STAT3 translocates to the nucleus and binds to the promoter region of IL-10-responsive genes, resulting in augmented transcription of M2c macrophage-associated genes^31,33,34^.

Aged garlic extract (AGE) has been shown to demonstrate various pharmacological actions including antihypertensive^35–37^, cardiovascular protective^38,39^, and immunomodulatory effects^40,41^. It is prepared by aging raw garlic (*Allium sativum* L.) in a water–ethanol mixture for more than 10 months at room temperature^42,43^. AGE contains several water-soluble sulfur compounds such as *S*-1-propenylcysteine (S1PC)^42,43^. Recently, S1PC has been shown to inhibit IL-6 production through autophagy activation, improve peripheral blood circulation by increasing the NOx production, and maintain endothelial barrier function^44–46^. We have previously reported that AGE inhibits lipid deposition in apolipoprotein E knockout (ApoE-KO) mice, an atherosclerosis model^47,48^. However, the underlying molecular mechanism and active constituents by which AGE improves atherosclerosis in vivo have not yet been identified.

Here, we aimed to explore the mechanism underlying the anti-atherosclerotic effect of AGE.

## Results

### AGE inhibited the formation of atherosclerotic plaques and the M1/M2-like macrophage ratio in ApoE-KO mice

**Figure 1:**
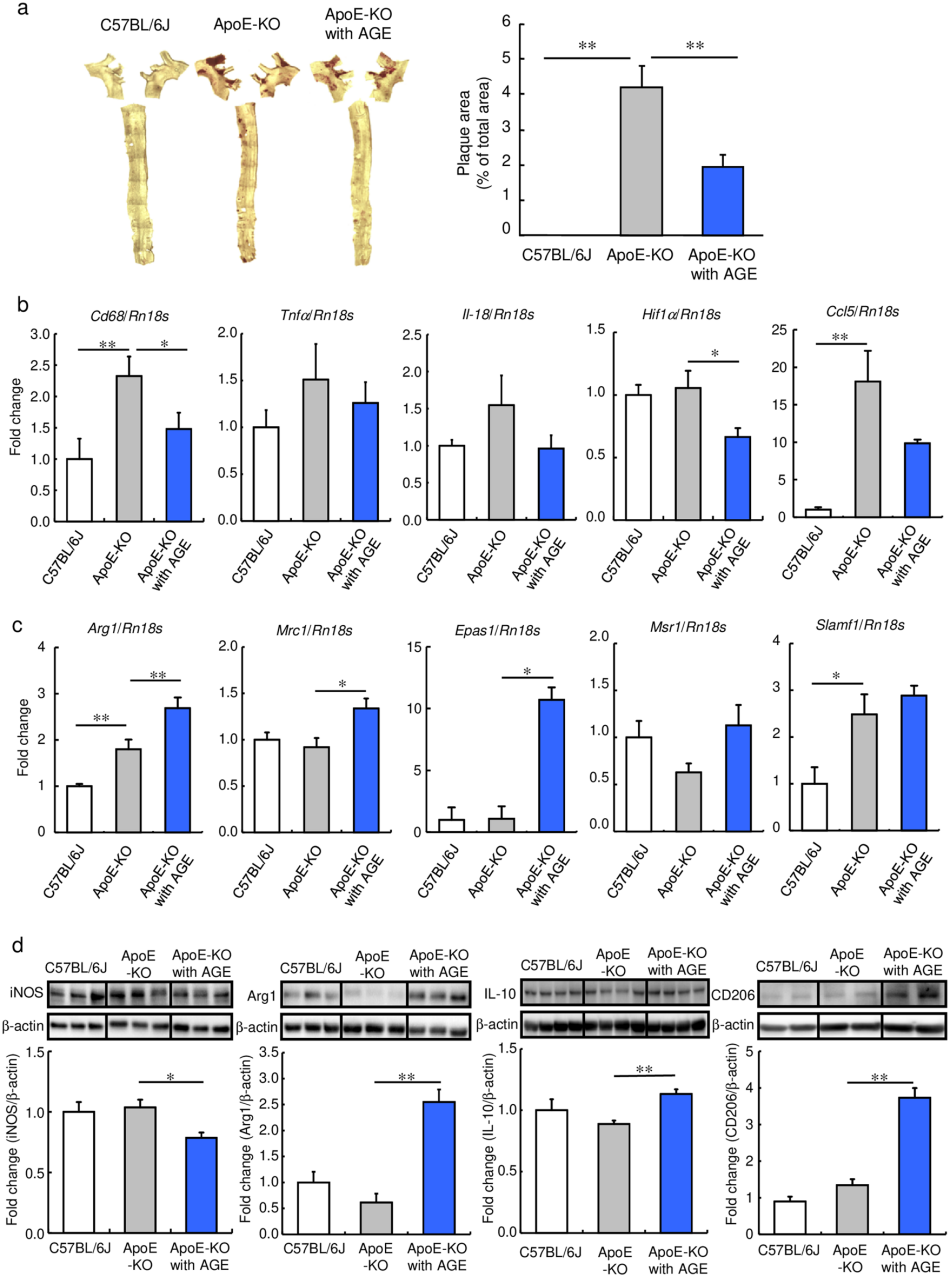
Effect of AGE on lipid deposition and M1/M2-macrophage marker expression in vascular tissue and spleen of ApoE-KO mice. C57BL/6 J (Control) and ApoE-KO mice were fed a diet with or without 3% AGE for 17 weeks. (**a**) Oil Red O staining of a thoracic aorta. Bar graphs show the percentage of the Oil Red O-positive area in the aorta. (**b**, **c**) The relative mRNA levels of (**b**) M1 macrophage marker genes (*Cd68*, *Tnfα*, *Il-18*, *Hif1α*, and *Ccl5*) and (**c**) M2 macrophage marker genes (*Arg1*, *Mrc1*, *Epas1*, *Msr1*, and *Slamf1*) in aorta. (**d**) The relative protein levels of iNOS, Arg1, IL-10, and CD206 in splenic lymphocytes. Data were quantified by analyzing each band intensity relative to a β-actin band after immunoblotting. The full-length blots are shown in Supplemental Figs. S8 and S9. Data are shown as mean + SEM (n = 5–8/group). Statistical differences were determined by Bonferroni’s multiple comparison test (*p < 0.05 and **p < 0.01).

To confirm the anti-atherosclerotic effect of AGE, we evaluated lipid accumulation in the vascular tissue of ApoE-KO mice fed a standard diet containing 3% AGE for 17 weeks. As shown in Fig. 1a, Supplemental Fig. S1a and b, and Table S1, AGE decreased the Oil Red O-positive areas in the aorta of the ApoE-KO mice compared with those of the mice fed normal diets, without altering the systemic lipid profiles.

We then investigated whether AGE affected macrophage polarization. Immunohistological staining showed that AGE reduced the number of macrophages expressing iNOS, an M1 macrophage marker, in the atherosclerotic lesions of the mice (Supplemental Fig. S1d). In addition, AGE significantly suppressed the levels of *Cd68* and *Hif1α* mRNA, out of five M1 macrophage marker genes (*Cd68*, *Tnfα*, *Il-18*, *Hif1α*, and *Ccl5*), in the whole aorta (Fig. 1b). Furthermore, AGE also reduced the protein level of inducible nitric oxide synthase (iNOS) in the spleen of the mice (Fig. 1d). On the other hand, AGE increased the number of macrophages expressing Arg1, an M2 macrophage marker, in the atherosclerotic lesions of the mice (Supplemental Fig. S1e). Moreover, out of five M2 macrophage marker genes (*Arg1*, *Mrc1* coding CD206, *Epas1* coding HIF2α, *Msr1* coding SR-AI, and *Slamf1* coding CD150), AGE significantly increased the levels of *Arg1, Mrc1 and Epas1* mRNA in the whole aorta (Fig. 1c). It also elevated the levels of Arg1, IL-10, and CD206 proteins in the spleen of the mice (Fig. 1d). These results suggested the possibility that AGE retards the progression of atherosclerosis partly through altering the M1/M2 macrophage ratio in several tissues.

### S1PC expanded the population of IL-10-induced M2c-like macrophages

IL-10 induces the expression of Arg1, resulting in the polarization of macrophages to M2c macrophages^49,50^. Since AGE increased the expression of Arg1 and IL-10 in ApoE-KO mice, we evaluated the effect of AGE on IL-10-induced increase in *Arg1* mRNA level in macrophage colony-stimulating factor (M-CSF)-induced bone marrow-derived macrophages (BMDMs). We found that AGE significantly enhanced the level of *Arg1* mRNA in recombinant mouse IL-10 (mIL-10)-treated BMDMs but not without mIL-10 (Supplemental Fig. S2a).

**Figure 2:**
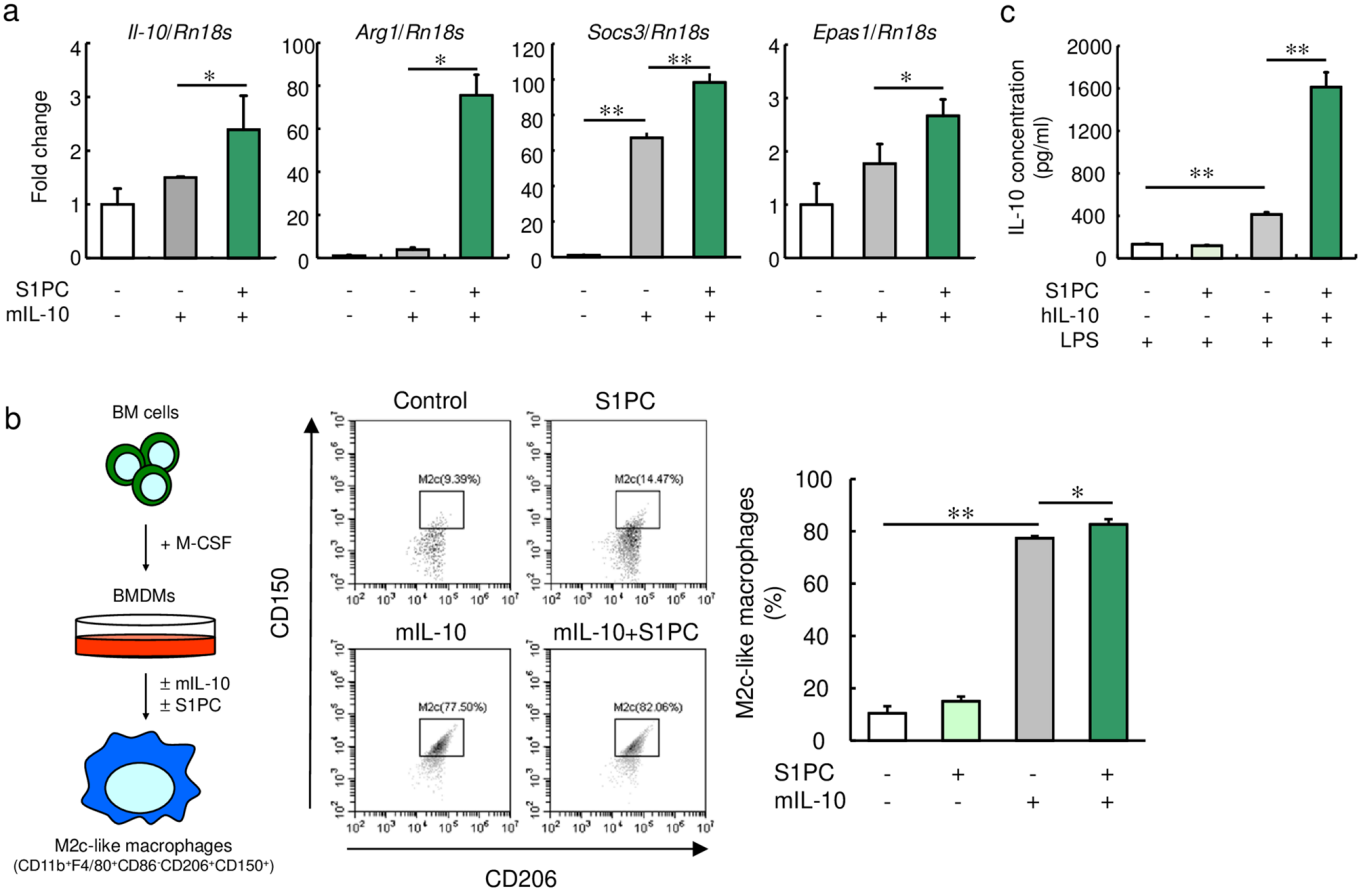
Effect of S1PC on IL-10-induced macrophage polarization in M-CSF-induced BMDMs. (**a**) M-CSF-induced BMDMs were treated with S1PC (300 µM) in the presence of mIL-10 (20 ng/mL) for 48 h. The relative levels of M2 macrophage marker genes (*Il-10*, *Arg1*, *Socs3*, and *Epas1*) were analyzed using qRT-PCR. (**b**) M-CSF-induced BMDMs were treated with S1PC (300 µM) in the presence or absence of mIL-10 (20 ng/mL) for 48 h. The population of M2c-like macrophages (CD11b^+^, F4/80^+^, CD86^-^, CD206^+^, and CD150^+^ cells) were analyzed by flow cytometry. (**c**) M-CSF-induced BMDMs were treated with S1PC (300 µM) in the presence or absence of hIL-10 (20 ng/mL) for 48 h. Then, they were washed with PBS and treated with LPS (50 ng/mL) for 6 h. The amounts of IL-10 secreted into the culture media were determined using ELISA. Data are shown as mean + SD. Data are representative of three independent experiments. Statistical differences were determined using Bonferroni’s multiple comparison test (*p < 0.05 and **p < 0.01).

We then examined the effect of S1PC (Supplemental Fig. S2b) on the expression of four M2 macrophage maker genes in mIL-10-treated BMDMs. As shown in Fig. 2a, Supplemental Fig. S2c and d, S1PC upregulated the levels of *Il-10*, *Arg1, Socs3,* and *Epas1* mRNA in the mIL-10-treated BMDMs, whereas S1PC had no effect on the expression of these mRNAs without mIL-10. Next, we assessed whether S1PC promoted the polarization of macrophages to M2c-like macrophages and found that S1PC increased the population of M2c-like macrophages (CD11b^+^, F4/80^+^, CD86^-^, CD206^+^, and CD150^+^ cells) when treated with mIL-10 for 48 h, but not 24 h and without mIL-10 (Fig. 2b and Supplemental Fig. S2e). These results suggest that S1PC is a major active constituent in AGE that promotes M2c-like macrophage polarization.

Distinctly polarized macrophages respond differently to toll-like receptor (TLR) 4 agonist lipopolysaccharides (LPS), such that M1 macrophages produce IL-12 and M2 macrophages produce IL-10^51^. To evaluate the anti-inflammatory ability of S1PC-induced M2c-like macrophages, we measured IL-10 production in M-CSF-induced BMDMs stimulated by LPS after treatment with recombinant human IL-10 (hIL-10) and S1PC. As shown in Fig. 2c, the BMDMs treated with S1PC and hIL-10 increased the LPS-induced IL-10 production compared with the macrophages treated with hIL-10 alone whereas S1PC-treated BMDMs without hIL-10 did not enhance IL-10 production induced by LPS. These results suggest that S1PC-treated M2c-like macrophages demonstrated high IL-10-producing abilities.

**Figure 3:**
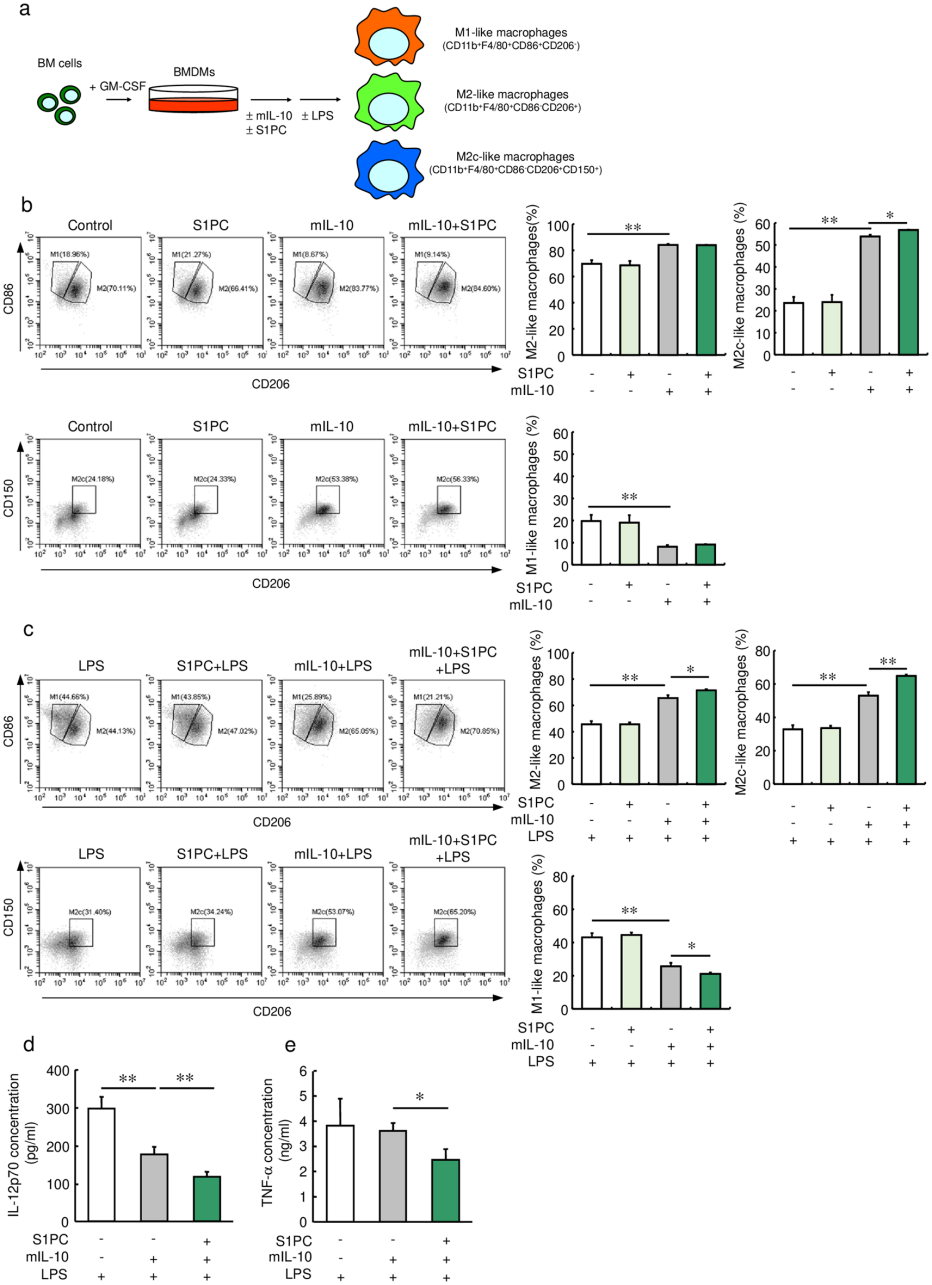
Effect of S1PC on IL-10-induced macrophage polarization in GM-CSF-induced BMDMs. (**a**) Assay scheme. (**b**) GM-CSF-induced BMDMs were treated with S1PC (300 µM) in the presence or absence of mIL-10 (30 ng/mL) for 48 h. The populations of M1-like macrophages (CD11b^+^, F4/80^+^, CD86^+^, and CD206^-^ cells), M2-like macrophages (CD11b^+^, F4/80^+^, CD86^-^, and CD206^+^ cells), and M2c-like macrophages were analyzed using flow cytometry. (**c**–**e**) GM-CSF-induced BMDMs were treated with S1PC (300 µM) in the presence or absence of mIL-10 (30 ng/mL) for 48 h. Then, they were washed with PBS and treated with LPS (100 ng/mL) for 16 h. (**c**) The populations of M1-like macrophages, M2-like macrophages, and M2c-like macrophages were analyzed using flow cytometry. The amounts of (**d**) IL-12p70 and (**e**) TNF-α secreted into the culture media were determined using ELISA. Data are shown as mean + SD. Data are representative of three independent experiments. Statistical differences were determined using Bonferroni’s multiple comparison test (*p < 0.05 and **p < 0.01).

Next, we investigated the effect of S1PC on changes in M1/M2 macrophage polarization by examining a population of granulocyte–macrophage colony-stimulating factor (GM-CSF)-induced BMDMs treated with S1PC in the absence or presence of mIL-10 (Fig. 3a, b). The BMDMs treated with mIL-10 exhibited significantly increased populations of M2c- and M2-like macrophages (CD11b^+^, F4/80^+^, CD86^-^, and CD206^+^ cells) and decreased population of M1-like macrophages (CD11b^+^, F4/80^+^, CD86^+^, and CD206^-^ cells) (Fig. 3b). In addition, S1PC treatment expanded the population of M2c-like macrophages in the presence of mIL-10 compared with BMDMs treated with mIL-10 alone. On the other hand, S1PC had no effect on the populations of M1- and M2-like macrophages in the absence or presence of mIL-10 (Fig. 3b). As shown in Fig. 3c, mIL-10 treated BMDMs had a higher percentage of M2-like macrophages, especially M2c-like macrophages, than M1-like macrophages in the presence of LPS, whereas BMDMs without mIL-10 showed a similar ratio of M1- to M2-like macrophages. In addition, S1PC and mIL-10 treated BMDMs was presented a high percentage of M2c-like macrophages and a low percentage of M1-like macrophages compared with mIL-10 alone. Furthermore, the S1PC and mIL-10-treated macrophages also exhibited greater reductions in the production of IL-12p70 (Fig. 3d) and TNF-α (Fig. 3e) induced by LPS compared with the cells treated with mIL-10 alone. These results suggest the possibility that S1PC promotes IL-10 production via increasing the population of M2c-like macrophages and reduces the population of M1-like macrophages.

### S1PC prolonged the activation and nuclear localization of STAT3 by inhibiting the interaction between IL-10Rα and SHIP1

**Figure 4:**
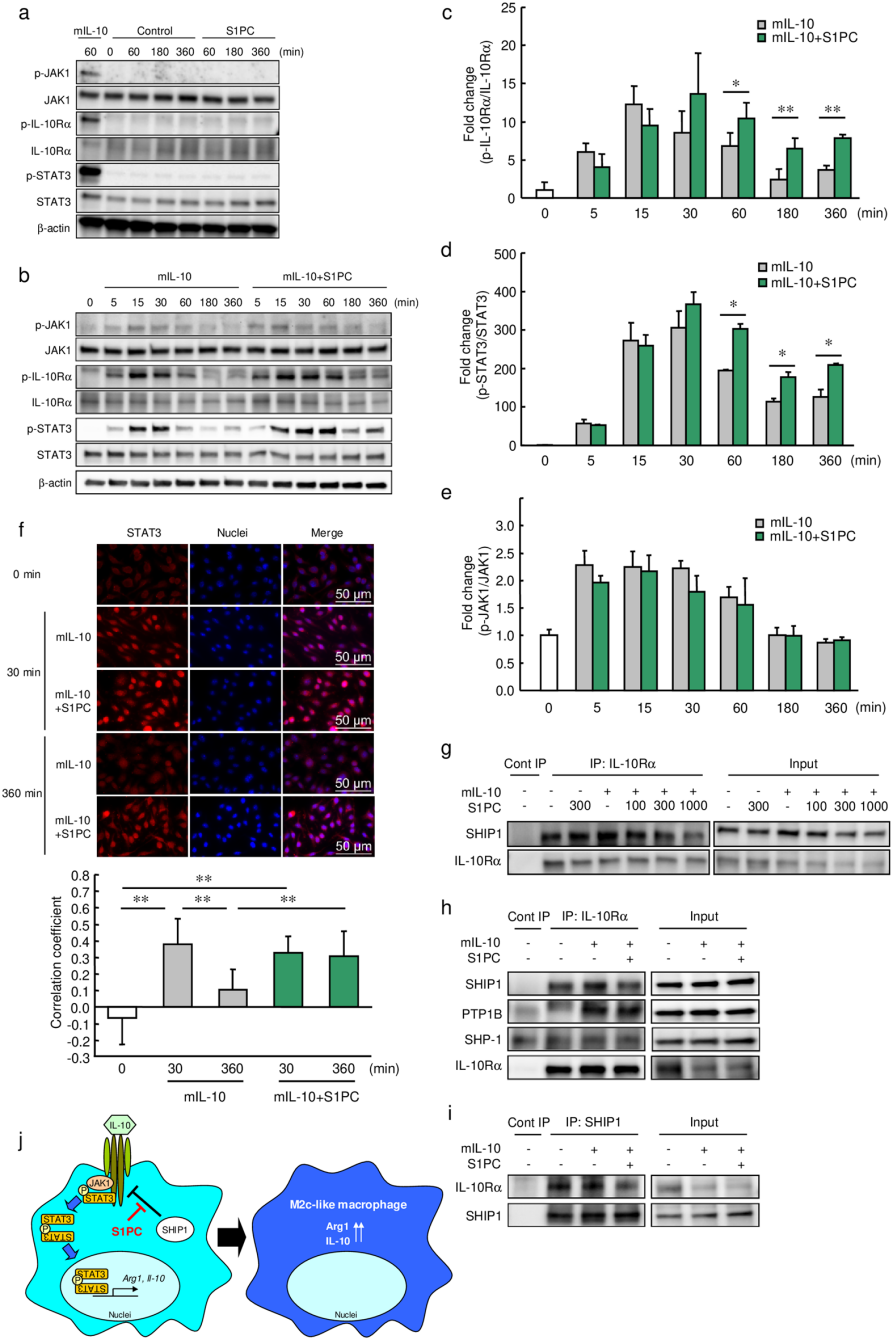
Effect of S1PC on the IL-10 signaling pathway. (**a**) M-CSF-induced BMDMs were treated with mIL-10 (2 ng/mL), PBS (Control) or S1PC (300 µM) for 60–360 min. The cell lysates were analyzed using immunoblotting with the indicated antibodies. The full-length blots are shown in Supplemental Fig. S10. (**b**–**e**) M-CSF-induced BMDMs were treated with S1PC (300 µM) in the presence of mIL-10 (2 ng/mL) for 5–360 min. The relative protein levels of (**c**) phospho-IL-10Rα, (**d**) phospho-STAT3, and (**e**) phospho-JAK1 was quantitated using immunoblotting. The full-length blots are shown in Supplemental Figs. S11 and S12. (**f**) M-CSF-induced BMDMs were immunostained with anti-STAT3 antibody (red) and hoechst 33342 (blue) for nuclear visualization after stimulation with mIL-10 (2 ng/mL) in the presence of S1PC (300 µM) for 30 and 360 min. Scale bar, 50 µm. Bar graphs show the correlation coefficients between nuclei and STAT3-positive regions. (**g**) J774A.1 cells were treated with S1PC (100–1000 µM) in the presence or absence of mIL-10 (10 ng/mL) for 1 h. Cell lysates were immunoprecipitated with anti-IL-10Rα antibody and analyzed using immunoblotting with the indicated antibodies. The full-length blots are shown in Supplemental Fig. S13. (**h**, **i**) J774A.1 cells were treated with S1PC (300 µM) in the presence of mIL-10 (10 ng/mL) for 1 h. Cell lysates were immunoprecipitated with (**h**) anti-IL-10Rα or (**i**) anti-SHIP1 antibodies and analyzed using immunoblotting with the indicated antibodies. The full-length blots are shown in Supplemental Figs. S14 and S15. (**j**) The proposed mechanism of macrophage polarization promoted by S1PC under M2c polarizing conditions. Data are shown as mean + SD. Data are representative of three independent experiments. Statistical differences were determined using (**f**) Bonferroni’s multiple comparison test or (**c**–**e**) Student’s *t*-test (*p < 0.05 and **p < 0.01).

The IL-10/IL-10R signaling cascade is the main pathway that controls macrophage polarization to the M2c phenotype^12^. To investigate the effect of S1PC on this signaling cascade, we measured the phosphorylation levels of JAK1, IL-10Rα, and STAT3 in M-CSF-induced BMDMs treated with or without mIL-10. S1PC had no effect on the phosphorylation of IL-10Rα, JAK1 and STAT3 without mIL-10 (Fig. 4a). As shown in Fig. 4b–d and Supplemental Fig. S4a, S1PC and AGE treatment prolonged the elevated levels of mIL-10-induced IL-10Rα and STAT3 phosphorylation for up to 360 min. The activation of IL-10Rα and STAT3 by S1PC were found to be dependent on IL-10 stimulation. In addition, STAT3 was localized to the nucleus at 30 min after mIL-10 treatment with or without S1PC. However, its nuclear localization continued up to 360 min only in S1PC treatment (Fig. 4f). On the other hand, S1PC did not affect mIL-10-induced JAK1 phosphorylation (Fig. 4b, e). In addition, S1PC did not alter the extent of phosphorylation of STAT3, STAT1, and STAT6 induced by IL-6, LPS with IFN-γ, and IL-4, respectively, in M-CSF-induced BMDMs, indicating that S1PC specifically affects IL-10-mediated IL-10R/STAT3 signaling (Supplemental Fig. S4b–d).

In the present study, S1PC prolonged the mIL-10-induced phosphorylation of IL-10Rα without affecting the phosphorylation of JAK1. Thus, we evaluated the effect of S1PC on the interaction between IL-10Rα and protein phosphatases through an immunoprecipitation assay. As shown in Fig. 4g, S1PC suppressed the interaction of IL-10Rα with SH2-containing inositol 5′-phosphatase 1 (SHIP1) in a concentration-dependent manner in J774A.1 cells in the presence of mIL-10 but not in the absence of mIL-10. On the other hand, S1PC could not suppress the interplay between IL-10Rα with protein tyrosine phosphatase 1B (PTP1B) or SH2-containing protein tyrosine phosphatase-1 (SHP-1) (Fig. 4h, i). In addition, S1PC had no effect on the protein levels of these phosphatases (Supplemental Fig. S5). Moreover, we found that the phosphorylation of IL-10Rα induced by mIL-10 was sustained in SHIP1-knockdown cells compared with control cells (Supplemental Fig. S6). These results suggest that S1PC treatment prolonged the IL-10-induced phosphorylation of IL-10Rα and STAT3 by suppressing the interaction between IL-10Rα and SHIP1, resulting in prolonged nuclear localization of STAT3 (Fig. 4j).

### S1PC increased the population of M2c-like macrophages in the senescence-accelerated mice

**Figure 5:**
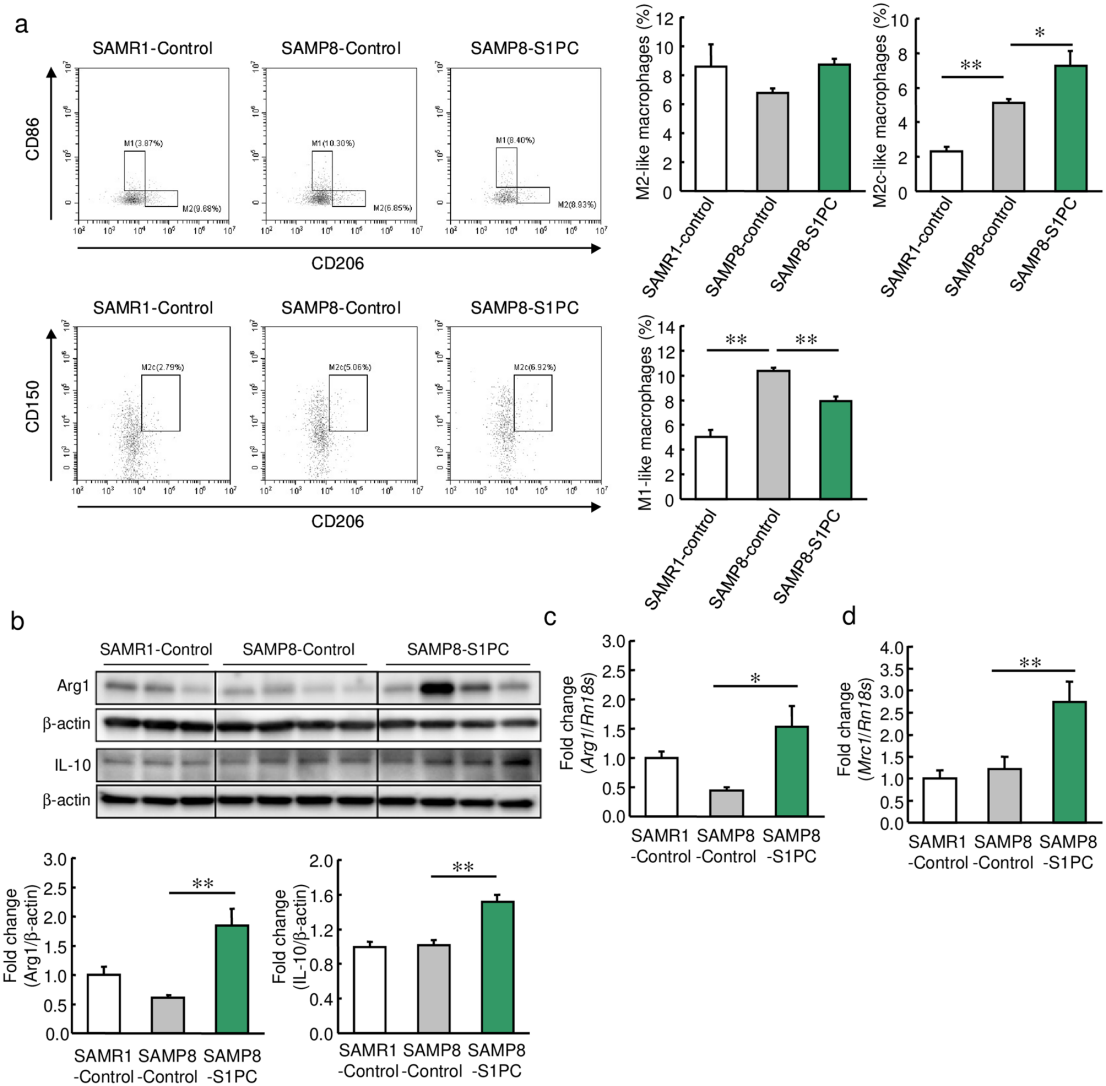
Effect of S1PC on macrophage polarization in SAMP8 mice. SAMR1 and SAMP8 mice were orally administered water and S1PC (5 mg/kg/day) for 6 weeks. (**a**) The populations of polarized macrophages in the splenic lymphocytes obtained from the SAMR1 and SAMP8 mice were analyzed using flow cytometry. Bar graphs show the populations of M1-like macrophages, M2-like macrophages, and M2c-like macrophages. (**b**) The relative protein levels of Arg1 and IL-10 in the splenic lymphocytes obtained from the SAMR1 and SAMP8 mice were determined using immunoblotting. The full-length blots are shown in Supplemental Fig. S16. (**c**, **d**) The relative mRNA levels of (**c**) *Arg1* and (**d**) *Mrc1* in the aortas were determined using qRT-PCR. Data are shown as mean + SEM (n = 4–8/group). Statistical differences were determined using Bonferroni’s multiple comparison test (*p < 0.05 and **p < 0.01).

Aging is a risk factor for atherosclerosis, and in aging tissues, macrophages shift to the M1 phenotype due to increased production of pro-inflammatory cytokines^52^. Thus, we examined whether S1PC shifted macrophage polarization in senescence-accelerated mouse prone 8 (SAMP8) mice. The population of M2c-like macrophages was significantly increased in the SAMP8 mice compared with senescence accelerated-resistant 1 (SAMR1) mice, and the population of M2c-like macrophages was further increased in the group treated with S1PC (Fig. 5a). In the SAMP8 mice, S1PC increased the protein levels of Arg1 and IL-10 in the spleen (Fig. 5b) as well mRNA levels of *Arg1* and *Mrc1* in the aorta (Fig. 5c, d). On the other hand, M1-like macrophages were found to be increased in the SAMP8 mice compared with the SAMR1 mice. However, S1PC significantly decreased the numbers of M1-like macrophages in the SAMP8 mice (Fig. 5a). Furthermore, S1PC enhanced the phosphorylation of STAT3 in the spleen of the SAMP8 mice at 60 min after oral administration of S1PC (Supplemental Fig. S7). These results suggest that S1PC promotes M2c-like macrophage polarization in senescent mice, regardless of the presence of atherosclerosis.

## Discussion

While AGE contains various bioactive constituents, it is unclear which constituents are involved in the increase of M2 macrophages. Since S1PC, one of active constituents in AGE, has been reported to demonstrate various pharmacological effects, such as improving barrier function^46^, peripheral blood flow^45^ and immunoregulatory function^44^, we hypothesized that it was of interest to examine whether S1PC increased M2-like macrophages induced by mIL-10. In the present study, we found that AGE and its constituent S1PC altered the balance between pro-inflammatory and anti-inflammatory macrophage populations by promoting M2-like macrophage polarization in the aorta of ApoE-KO and SAMP8 mice.

In the atherosclerotic plaques of LDL receptor-KO mice, M1 macrophages predominate and account for approximately 40%, while M2 macrophages account for about 20% of the total number of macrophages^53^. M1 macrophages were found to accelerate the formation of atherosclerotic plaques, while M2 macrophages were found to contribute to the suppression of both inflammation and plaque formation in an atherosclerosis mouse model^54–56^. Thus, the balance between the M1 and M2 macrophages play an important role in the formation and regression of atherosclerotic plaques.

Previous reports have shown that the Arg1/iNOS ratio is a key determinant in M1/M2 macrophage polarization^57,58^. Arg1 is an enzyme that catalyzes the conversion of L-arginine to ornithine and urea, competing with the M1 macrophage marker-enzyme iNOS which utilizes L-arginine^57,58^. It has been shown that the levels of iNOS and IL-6 are high in Arg1-deficient macrophages and low in Arg1-overexpressed macrophages, leading to reduced numbers of M1 macrophages^57,58^. The increase in M2 macrophages induced by AGE and S1PC may be attributable to the enhanced expression of Arg1 in the aorta, spleen, and BMDMs of mice.

The transcription of the *Arg1* gene is induced by direct binding of STAT3 to the promoter region^59^. Furthermore, the phosphorylation of STAT3 is a critical step in IL-6R and IL-10R signaling that determines the switching between pro- and anti-inflammatory gene expression induced by IL-6 or IL-10^60^. Therefore, the prolongation of STAT3 phosphorylation and nuclear localization by S1PC may enhance the level of *Arg1* mRNA. On the other hand, the expression of Arg1 is also induced by the activation of STAT6 via IL-4 treatment, which then induces the formation of M2a macrophages. We found that S1PC had no effect on the IL-4-induced phosphorylation of STAT6, suggesting that it does not affect the expression of Arg1 and the polarization of macrophages to M2a macrophages. S1PC was observed to affect only M2c-like macrophages polarization but not M2a macrophages by promoting the IL-10Rα-STAT3 signaling pathway.

Since S1PC prolonged the mIL-10-induced phosphorylation of IL-10Rα and STAT3, we hypothesized that S1PC may regulate the dephosphorylation of IL-10Rα through protein phosphatases. Several SH2 domain-containing protein phosphatases, including SHP-1, PTP1B, and SHIP1, have been shown to negatively regulate the JAK/STAT signaling pathway^61–64^. Thus, we also hypothesized that the prolongation of IL-10Rα phosphorylation was due to the inhibition of protein phosphatases against IL-10Rα. We found that SHIP1 interacted with IL-10Rα, and its interaction was inhibited by S1PC. Since knockdown of SHIP1 extended IL-10Rα activation, S1PC may sustain the phosphorylated state of IL-10Rα by blocking the interaction with SHIP1 and IL-10Rα. On the other hand, it has been reported that SHIP1 is important for IL-10R signaling because it interacts with STAT3 and translocates STAT3 to the nuclei^65^. It is likely that S1PC does not inhibit the interaction between STAT3 and SHIP1 because it sustains the nuclear localization of STAT3.

While AGE and S1PC directly promoted the polarization of macrophages to M2c macrophages, they may also have influenced their polarization to M1 macrophages. M2 macrophages produce IL-10 and TGF-β, which exert anti-inflammatory and tissue repair actions^66^. While IL-10 promotes polarization of macrophages to M2c macrophages by inducing phosphorylation of STAT3, it also suppresses their polarization to M1 macrophages and the production of pro-inflammatory cytokines through inhibition of STAT1 and NF-κB^67^. Since AGE and S1PC were observed to increase the production of IL-10 in the spleen with increased expressions of M2 macrophage marker proteins, they likely increased IL-10 production in the aorta, as well as observed by the increases in M2 macrophages. Therefore, M1 macrophage polarization reduced by AGE and S1PC may also be due to the increased IL-10 production by the expanded numbers of M2 macrophages. However, S1PC notably did not affect the IL-6-induced phosphorylation of STAT3 and LPS plus IFN-γ-induced phosphorylation of STAT1, suggesting that S1PC does not directly inhibit M1 macrophage polarization through the polarization signaling pathway.

Inflammaging refers to chronic low-grade inflammation that occurs with aging and is a risk factor for atherosclerosis. Inflammaging is promoted by persistent increases in CRP and pro-inflammatory cytokines such as IL-6 and TNF-α^68–74^. The augmented release of DAMPs from damaged tissues and dead cells triggers an increase in pro-inflammatory cytokines, promoting inflammaging in vascular tissues. In addition, vascular aging enhances the secretion of SASP factors^75,76^. Both DAMPs and SASP factors cause the polarization of macrophages to the M1 phenotype in various tissues, inducing further production of pro-inflammatory cytokines and leading to a feedback loop of inflammaging^9,77^. Moreover, activation of the TLR signaling pathway by DAMPs has been reported to increase the shift of M2 macrophages to the M1 phenotype^78,79^. In this study, we found that S1PC increased the percentage of IL-10-induced M2c-like macrophages and decreased the percentage of M1-like macrophages upon LPS stimulation. In addition, the reduction of M1-like macrophage polarization by S1PC inhibited inflammatory cytokine production. It is possible that the macrophages polarized by S1PC may inhibit DAMPs-induced macrophage polarization to M1-like macrophages. It was observed in several tissues from the SAMP8 mice that the increases in M1 macrophages and decreases in M2 macrophages resulted in enhanced inflammaging^52,80,81^. Additionally, S1PC sustained the phosphorylation of STAT3, increased the population of M2c-like macrophages, and reduced the population of M1-like macrophages. This S1PC-induced polarization shift from M1- to M2c-like macrophages occurred not only in the spleen, but also in the aorta of the mice. These findings suggest that S1PC may suppress inflammaging in the whole body by exerting anti-inflammatory effects due to the increased M2c/M1 macrophage ratio.

In this study, we presented evidence indicating that AGE inhibits the formation of atherosclerotic plaque and increased the M2/M1 macrophage ratio in the aorta. Additionally, we found that S1PC is the major component of AGE responsible for its anti-atherosclerotic and anti-inflammatory effects. S1PC has been reported to have anti-inflammatory effect by degrading myeloid differentiation factor 88, a crucial adaptor protein of TLRs^44^. Our findings in this study showed that anti-inflammatory effect of S1PC was mediated via another mechanism, suggesting that S1PC is a compound that produces various effects through different targets. In addition, since M2 macrophages are involved in the inhibition of various chronic inflammatory diseases such as diabetes, chronic kidney disease, autoimmune diseases, and atherosclerosis^82–84^, AGE and S1PC may serve as a potentially beneficial agent for these diseases.

## Methods

### Preparation of AGE and S1PC

AGE was manufactured as follows: raw garlic (*Allium sativum L*.) was sliced, immersed in aqueous ethanol (20–50%), and incubated for more than 10 months at room temperature. The specific procedure and specifications are in compliance with the US Pharmacopeia/Natural Formula (USP/NF) for garlic fluid extract monograph^85^. S1PC was synthesized as previously described^44,86,87^ and the purity was > 99.8%.

### Antibodies and reagents

The antibodies used in this study are summarized in Supplemental Table S2. Recombinant mouse M-CSF (#576404), IL-10 (#575806) and IL-6 (#575702) were obtained from Biolegend. Recombinant mouse GM-CSF (#ab198564) was purchased from Abcam. Recombinant human IL-10 (#200-10) was obtained from PeproTech. Recombinant mouse IFN-γ (#21-8311) and IL-4 (#21-8041) were purchased from TONBO biosciences. Oil Red O (#O0625) and LPS (#L2630) were obtained from Sigma-Aldrich.

### Animals

Male C57BL/6 J mice and ApoE-KO mice (B6.129P2-*Apoe*^*tm1Unc*^/J, 5 weeks old) were purchased from Charles River Japan. Male SAMR1 and SAMP8 mice (5 weeks old) were purchased from Japan SLC. The animals were individually housed under a 12 h light–dark cycle with controlled temperature (22 ± 1 °C) and humidity (50 ± 5%) and had free access to food and water. The experiments were performed in accordance with protocols approved by the Wakunaga Pharmaceutical Company Institutional Animal Care and Use Committee (Permission No. 252 and 290). The procedures performed in this investigation conform to the Guide for the Care and Use of Laboratory Animals published by the US National Institute of Health^88^. The study was performed in accordance with ARRIVE guidelines.

### Quantitation of atherosclerosis

C57BL/6 J (6 weeks old, n = 5/group) and ApoE-KO mice (6 weeks old, n = 8/group) were fed standard diets with or without 3% AGE (CLEA Japan) for 17 weeks. Then, their plasma, hearts, aortas, and spleens were collected. Plasma levels of triglyceride (TG), total cholesterol (TC), high-density lipoprotein cholesterol (HDL-C), and non-esterified fatty acid (NEFA) were quantified using commercially available kits (#432-40201, #439-17501, #431-52501, and #279-75401, respectively; FUJIFILM Wako Pure Chemical). The aortas were stained with Oil Red O solution for 20 min then washed with isopropanol and ultrapure water. The stained areas were analyzed using BZ-9000 (KEYENCE) and quantified using ImageJ software version 1.48 (National Institutes of Health).

### Immunohistological staining

The upper half of the hearts were embedded in paraffin, and the aortic roots were sectioned at thicknesses of 4 µm. The sections were then deparaffinized and incubated with proteinase K (#34060-96, KANTO KAGAKU) at 37 °C for 30 min for antigen activation. After washing with PBS, the sections were blocked with PBS containing 5% BSA for 1 h at room temperature (RT). They were then incubated with primary antibodies at 4 °C overnight and subsequently incubated with anti-rabbit Alexa Fluor 488 and anti-actin-555 Phalloidin (#PHDH1, Cytoskeleton) for 1 h at RT. After washing with PBS, they were mounted with DAPI-containing mounting medium. The stained sections were analyzed under a fluorescence microscope (Axio Observer7) using ZEN 3.1 pro software (ZEISS).

### Evaluation of macrophage polarization and STAT3 phosphorylation in vivo

For the evaluation of macrophage polarity changes, SAMR1 and SAMP8 mice (8 weeks old) were divided into the 3 groups (n = 6 or 8/group) and orally administered water or S1PC (5 mg/kg, dissolved in water) once-daily for 6 weeks. Their aortas and spleens were collected after blood sampling. Macrophage polarization in the splenic lymphocytes was analyzed using flow cytometry. Protein and mRNA levels of M1/M2 macrophage markers were evaluated in the splenic lymphocytes and aortas of the mice using immunoblotting and qRT-PCR. For the experiment to examine STAT3 phosphorylation, SAMP8 mice (10 weeks old) were randomized into 5 groups (n = 5/group). These mice received single oral administration of S1PC (5 mg/kg, dissolved in water) and they were sacrificed after 15–180 min. Their spleens were collected and the splenic lymphocytes were subjected to immunoblotting.

### Preparation of bone marrow-derived macrophages (BMDMs)

Bone marrow cells were collected from femurs and tibiae of C57BL/6 J mice (6–10 weeks old). Cells (5 × 10^5^ cells/mL) were cultured in differentiation medium (RPMI1640 containing 10% FBS, 100 U/mL penicillin and 100 µg/mL streptomycin and 40 ng/mL M-CSF or 40 ng/mL GM-CSF) for 3 days, and then cultured for another 2 days after adding a half volume of differentiation medium. After removing non-adherent cells, adherent cells were cultured for another 2 days in the presence of 40 ng/mL M-CSF or 40 ng/mL GM-CSF.

### In vitro macrophage polarization

For macrophage polarization analysis under non-inflammatory conditions, M-CSF-induced BMDMs were treated with 20 ng/mL mIL-10 in the presence or absence of 300 µM S1PC at 37 °C for 24 or 48 h on temperature-responsive cell culture plates (DIC). For polarization analysis under inflammatory conditions, GM-CSF-induced BMDMs were treated with 30 ng/mL mIL-10 in the presence or absence of 300 µM S1PC at 37 °C for 48 h on temperature-responsive cell culture plates, and then incubated with 100 ng/mL LPS for 16 h. The cells were placed on ice for 15 min then collected via centrifugation (1000 g, 5 min, 4 °C). The macrophage phenotypes were evaluated using flow cytometry.

### Flow cytometry

BMDMs and splenic lymphocytes were treated with PBS containing 5% BSA and FcR blocking reagent (#130-092-575, Miltenyi Biotec) for 15 min at 4 °C. Then, cells were incubated with indicated antibodies for 30 min at 4 °C. The population of M1- (CD11b^+^, F4/80^+^, CD86^+^, and CD206^-^ cells), M2- (CD11b^+^, F4/80^+^, CD86^-^, and CD206^+^ cells) and M2c-like (CD11b^+^, F4/80^+^, CD86^-^, CD206^+^, and CD150^+^ cells) macrophage was analyzed by a CytoFLEX using CytExpert software version 2.0.0.153 (Beckman Coulter).

### Immunoblotting

M-CSF-induced BMDMs were cultured with mIL-10 (2 ng/mL), IL-6 (10 ng/mL), IL-4 (5 ng/mL) or IFN-γ (5 ng/mL), and LPS (25 ng/mL) in the presence of S1PC (300 µM) or AGE (2 mg/mL) at 37 °C at indicated time points. The spleens were finely crushed in HBSS containing 5% FBS using a plunger, and the red blood cells were lysed using BD Pharm lyse buffer. The splenic lymphocytes were passed through a 40 µm cell strainer and washed with HBSS containing 5% FBS. The cells were lysed in radioimmunoprecipitation assay (RIPA) lysis buffer (Merck Millipore) containing Halt protease and phosphatase inhibitor single-use cocktail (Thermo Fisher Scientific). The lysates were collected by centrifugation (12,000 g, 10 min, 4 °C), and the protein levels were determined using a BCA protein assay kit (#23225, Thermo Fisher Scientific). The lysates were then boiled in sample buffer solution at 95 °C. Immunoblotting analysis was performed as previously described^44,46^. The blotted membranes were incubated with the indicated first antibodies and then the secondary antibodies. Immunoreactive proteins were detected using an ECL Prime western blotting detection reagent (Cytiva) or Clarity Max Western ECL substrate (Bio-Rad), and visualized bands were analyzed on a ChemiDoc Touch MP Imaging System using Image Lab software version 6.0 (Bio-Rad).

### Quantitative real-time PCR (qRT-PCR)

Total RNA was extracted from BMDMs, splenic lymphocytes, and aortas using RNAiso reagent (Takara Bio), and cDNA was synthesized using PrimeScript RT reagent Kit with gDNA Eraser (#RR047, Takara Bio). The quantitative real-time PCR was performed using KAPA SYBR FAST qPCR Master Mix (KAPA Biosystems) or TaqMan Fast Advanced Master Mix (#4444556, Applied Biosystems) to determine the relative expression level of target genes based on the 18S ribosomal RNA gene (*Rn18s*) in a CFX96 Touch Real time PCR Detection System (Bio-Rad). The primer sequences are listed in Supplemental Table S3. The relative mRNA level was calculated using the comparative CT (ΔΔCT) method.

### Evaluation of STAT3 nuclear localization

M-CSF-induced BMDMs were seeded onto 8-well chamber slides and treated with mIL-10 (2 ng/mL) in the presence of S1PC (300 µM) at 37 °C for 30 or 360 min. Then, cells were fixed by 4% paraformaldehyde at 4 °C for 30 min and treated with PBS containing 5% BSA, 0.3% Triton X-100 and FcR blocking reagent at 4 °C for 15 min. Then, cells were incubated with the anti-STAT3 antibody at 4 °C for 30 min and were subsequently incubated with anti-mouse IgG-AlexaFluor 594 and Hoechst 33342 (#H3570, Thermo Fisher Scientific) at 4 °C for 30 min. Stained-cells were analyzed by a fluorescence microscope, Axio Observer7, using ZEN 3.1 pro software (ZEISS). Pearson’s correlation coefficient was measured as a statistic for quantifying colocalization of STAT3 and nuclei^89^.

### Immunoprecipitation

J774A.1 cells were maintained in RPMI1640 medium (10% FBS and 1% penicillin/streptomycin). Cells were seeded onto 10 cm dishes and treated with 10 ng/mL mIL-10 in the presence of S1PC (100, 300, and 1000 µM) for 1 h and were lysed by RIPA lysis buffer containing Halt protease and phosphatase inhibitors. Anti-IL-10Rα or anti-SHIP1 antibody was incubated with Dynabeads Protein G (#DB10003, Thermo Fisher Scientific) for 10 min at RT and then cell lysates were incubated with antibody-beads complexes for 1 h at RT. Beads were washed 3 times with PBS and boiled in sample buffer solution for 10 min at 70 °C. The samples were subjected to immunoblot analysis with the indicated antibodies.

### Knockdown of Inpp5d (SHIP1) gene with small interfering RNA (siRNA)

J774A.1 cells seeded on 12-well plates were transfected with 2.5 pmol control siRNA (#4390843, Silencer select negative control No. 1 siRNA, Thermo Fisher Scientific) or Inpp5d (SHIP1) siRNA (#4390771, Silencer select mouse Inpp5d siRNA, ID: s68357, Thermo Fisher Scientific) using INTERFERin reagent (#409-10, Polyplus transfection) according to the manufacturer’s instructions. After 72 h of the transfection, the cells were treated with mIL-10 (10 ng/mL) for 30–180 min. The samples were subjected to immunoblot analysis with the indicated antibodies.

### Cytokine measurements

For IL-10 production assay, M-CSF-induced BMDMs were cultured with 20 ng/mL hIL-10 in the presence or absence of 300 µM S1PC at 37 °C. After 48 h, cells were washed with pre-warmed PBS and then treated with 50 ng/mL LPS for 6 h. For IL-12p70 and TNF-α production assay, GM-CSF-induced BMDMs were cultured with 30 ng/mL mIL-10 in the presence or absence of 300 µM S1PC at 37 °C. After 48 h, cells were washed with pre-warmed PBS and then treated with 100 ng/mL LPS for 16 h. Culture supernatants were collected by centrifugation at 1000 g at 4 °C for 5 min. The level of each cytokine in the supernatant was determined by ELISA kit (#88-7105, #88-7121, and #88-7324, respectively, Thermo Fisher Scientific).

### Statistical analysis

All data were subject to outliers were eliminated using Thomson's rejection test. The statistical significance of differences was evaluated by student *t*-test or one-way analysis of variance (ANOVA) followed by Bonferroni’s multiple comparison test using WinSTAT statistics software version 1.2 (M. Sato, Japan). Difference at *p* < 0.05 was considered to be significant.

## Supplementary Information

Supplementary Information.

## References

[1] Falk E (2006). Pathogenesis of atherosclerosis. J. Am. Coll. Cardiol..

[2] Wolf D, Ley K (2019). Immunity and inflammation in atherosclerosis. Circ. Res..

[3] Das R (2013). Macrophage gene expression and foam cell formation are regulated by plasminogen. Circulation.

[4] Willemsen L, de Winther MPJ (2020). Macrophage subsets in atherosclerosis as defined by single-cell technologies. J. Pathol..

[5] Falck-Hansen M, Kassiteridi C, Monaco C (2013). Toll-like receptors in atherosclerosis. Int. J. Mol. Sci..

[6] Xu H (2019). Vascular macrophages in atherosclerosis. J. Immunol. Res..

[7] Barrett TJ (2020). Macrophages in atherosclerosis regression. Arterioscler. Thromb. Vasc. Biol..

[8] Ferrucci L, Fabbri E (2018). Inflammageing: chronic inflammation in ageing, cardiovascular disease, and frailty. Nat. Rev. Cardiol..

[9] Oishi Y, Manabe I (2016). Macrophages in age-related chronic inflammatory diseases. NPJ Aging Mech. Dis..

[10] Mills CD, Kincaid K, Alt JM, Heilman MJ, Hill AM (2000). M-1/M-2 Macrophages and the Th1/Th2 paradigm. J. Immunol..

[11] Mosser DM, Edwards JP (2008). Exploring the full spectrum of macrophage activation. Nat. Rev. Immunol..

[12] Rőszer T (2015). Understanding the mysterious M2 macrophage through activation markers and effector mechanisms. Mediators Inflamm..

[13] Yang S (2020). Macrophage polarization in atherosclerosis. Clin. Chim. Acta.

[14] Bobryshev YV, Ivanova EA, Chistiakov DA, Nikiforov NG, Orekhov AN (2016). Macrophages and their role in atherosclerosis: pathophysiology and transcriptome analysis. Biomed. Res. Int..

[15] Lee SG (2018). Macrophage polarization and acceleration of atherosclerotic plaques in a swine model. PLoS ONE.

[16] De Paoli F, Staels B, Chinetti-Gbaguidi G (2014). Macrophage phenotypes and their modulation in atherosclerosis. Circ. J..

[17] Leitinger N, Schulman IG (2013). Phenotypic polarization of macrophages in atherosclerosis. Arterioscler. Thromb. Vasc. Biol..

[18] Zizzo G, Hilliard BA, Monestier M, Cohen PL (2012). Efficient clearance of early apoptotic cells by human macrophages requires M2c polarization and MerTK induction. J. Immunol..

[19] Angsana J, Chen J, Liu L, Haller CA, Chaikof EL (2016). Efferocytosis as a regulator of macrophage chemokine receptor expression and polarization. Eur. J. Immunol..

[20] Nathan CF, Murray HW, Wlebe IE, Rubin BY (1983). Identification of interferon-γ, as the lymphokine that activates human macrophage oxidative metabolism and antimicrobial activity. J. Exp. Med..

[21] MacMicking J, Xie QW, Nathan C (1997). Nitric oxide and macrophage function. Annu. Rev. Immunol..

[22] Takeda N (2010). Differential activation and antagonistic function of HIF-a isoforms in macrophages are essential for NO homeostasis. Genes Dev..

[23] Donners MMPC (2016). Cathepsin K deficiency prevents the aggravated vascular remodeling response to flow cessation in ApoE-/- Mice. PLoS ONE.

[24] Benoit M, Desnues B, Mege J-L (2008). Macrophage polarization in bacterial infections. J. Immunol..

[25] Martinez FO, Sica A, Mantovani A, Locati M (2008). Macrophage activation and polarization. Front. Biosci..

[26] Ka MB, Daumas A, Textoris J, Mege J-L (2014). Phenotypic diversity and emerging new tools to study macrophage activation in bacterial infectious diseases. Front. Immunol..

[27] Kim D (2019). Ubiquitin E3 ligase pellino-1 inhibits IL-10-mediated M2c polarization of macrophages, thereby suppressing tumor growth. Immune Netw..

[28] Moore KW, De Waal Malefyt R, Coffman RL, O’Garra A (2001). Interleukin-10 and the interleukin-10 receptor. Annu. Rev. Immunol..

[29] Walter MR (2014). The molecular basis of IL-10 function: from receptor structure to the onset of signaling. Curr. Top. Microbiol. Immunol..

[30] Riley JK, Takeda K, Akira S, Schreiber RD (1999). Interleukin-10 receptor signaling through the JAK-STAT pathway. Requirement for two distinct receptor-derived signals for anti-inflammatory action. J. Biol. Chem..

[31] Shouval DS (2014). Interleukin 10 receptor signaling: master regulator of intestinal mucosal homeostasis in mice and humans. Adv. Immunol..

[32] Kotenko SV (1997). Identification and functional characterization of a second chain of the interleukin-10 receptor complex. EMBO J..

[33] Murray PJ (2006). Understanding and exploiting the endogenous interleukin-10/STAT3-mediated anti-inflammatory response. Curr. Opin. Pharmacol..

[34] Lobo-Silva D, Carriche GM, Castro AG, Roque S, Saraiva M (2016). Balancing the immune response in the brain: IL-10 and its regulation. J. Neuroinflamm..

[35] Ried K, Frank OR, Stocks NP (2013). Aged garlic extract reduces blood pressure in hypertensives: a dose-response trial. Eur. J. Clin. Nutr..

[36] Ried K, Travica N, Sali A (2016). The effect of aged garlic extract on blood pressure and other cardiovascular risk factors in uncontrolled hypertensives: the AGE at Heart trial. Integr. Blood Press. Control.

[37] Matsutomo T (2017). Metabolomic study on the antihypertensive effect of S -1-propenylcysteine in spontaneously hypertensive rats using liquid chromatography coupled with quadrupole-orbitrap mass spectrometry. J. Chromatogr. B.

[38] Budoff M (2006). Aged garlic extract retards progression of coronary artery calcification. J. Nutr..

[39] Matsumoto S (2016). Aged garlic extract reduces low attenuation plaque in coronary arteries of patients with metabolic syndrome in a prospective randomized double-blind study. J. Nutr..

[40] Nantz MP (2012). Supplementation with aged garlic extract improves both NK and γδ-T cell function and reduces the severity of cold and flu symptoms: a randomized, double-blind, placebo-controlled nutrition intervention. Clin. Nutr..

[41] Xu C (2018). Aged garlic extract supplementation modifies inflammation and immunity of adults with obesity: a randomized, double-blind, placebo-controlled clinical trial. Clin. Nutr. ESPEN.

[42] Kodera Y, Ushijima M, Amano H, Suzuki JI, Matsutomo T (2017). Chemical and biological properties of s-1-propenyl-l-cysteine in aged garlic extract. Molecules.

[43] Kodera Y, Kurita M, Nakamoto M, Matsutomo T (2019). Chemistry of aged garlic: diversity of constituents in aged garlic extract and their production mechanisms via the combination of chemical and enzymatic reactions (Review). Exp. Ther. Med..

[44] Suzuki J (2018). Anti-inflammatory action of cysteine derivative S-1-propenylcysteine by inducing MyD88 degradation. Sci. Rep..

[45] Ushijima M, Kunimura K, Suzuki J (2020). S-1-Propenylcysteine, a sulfur compound in aged garlic extract, alleviates cold-induced reduction in peripheral blood flow in rat via activation of the AMPK/eNOS/NO pathway. Exp. Ther. Med..

[46] Kunimura K, Miki S, Takashima M, Suzuki J (2021). S-1-propenylcysteine improves TNF-α-induced vascular endothelial barrier dysfunction by suppressing the GEF-H1/RhoA/Rac pathway. Cell Commun. Signal..

[47] Morihara N, Hino A, Yamaguchi T, Suzuki J (2016). Aged garlic extract suppresses the development of atherosclerosis in apolipoprotein E-knockout mice. J. Nutr..

[48] Morihara N, Hino A, Miki S, Takashima M, Suzuki J (2017). Aged garlic extract suppresses inflammation in apolipoprotein E-knockout mice. Mol. Nutr. Food Res..

[49] Jung M (2017). IL-10 improves cardiac remodeling after myocardial infarction by stimulating M2 macrophage polarization and fibroblast activation. Basic Res. Cardiol..

[50] Rath M, Müller I, Kropf P, Closs EI, Munder M (2014). Metabolism via arginase or nitric oxide synthase: two competing arginine pathways in macrophages. Front. Immunol..

[51] Chung S (2015). Distinct role of FoxO1 in M-CSF- and GM-CSF-differentiated macrophages contributes LPS-mediated IL-10: implication in hyperglycemia. J. Leukoc. Biol..

[52] Karuppagounder V (2016). Modulation of macrophage polarization and HMGB1-TLR2/TLR4 cascade plays a crucial role for cardiac remodeling in senescence-accelerated prone mice. PLoS ONE.

[53] De Paoli F, Staels B, Chinetti-Gbaguidi G (2014). Macrophage phenotypes and their modulation in atherosclerosis. Circ. J..

[54] Bi Y (2019). M2 macrophages as a potential target for antiatherosclerosis treatment. Neural Plast..

[55] Gong M, Zhuo X, Ma A (2017). STAT6 upregulation promotes M2 macrophage polarization to suppress atherosclerosis. Med. Sci. Monit. Basic Res..

[56] Wang G, Liu X, Li X, Zhao Y (2021). Suppression of PAPP-A mitigates atherosclerosis by mediating macrophage polarization via STAT3 signaling. Biochem. Biophys. Res. Commun..

[57] Suwanpradid J (2017). Arginase1 deficiency in monocytes/macrophages upregulates inducible nitric oxide synthase to promote cutaneous contact hypersensitivity. J. Immunol..

[58] El Kasmi KC (2008). Toll-like receptor-induced arginase 1 in macrophages thwarts effective immunity against intracellular pathogens. Nat. Immunol..

[59] Vasquez-Dunddel D (2013). STAT3 regulates arginase-i in myeloid-derived suppressor cells from cancer patients. J. Clin. Invest..

[60] Yasukawa H (2003). IL-6 induces an anti-inflammatory response in the absence of SOCS3 in macrophages. Nat. Immunol..

[61] Seif F (2017). The role of JAK-STAT signaling pathway and its regulators in the fate of T helper cells. Cell Commun. Signal..

[62] Morris R, Kershaw NJ, Babon JJ (2018). The molecular details of cytokine signaling via the JAK/STAT pathway. Protein Sci..

[63] Perez LE, Desponts C, Parquet N, Kerr WG (2008). SH2-inositol phosphatase 1 negatively influences early megakaryocyte progenitors. PLoS ONE.

[64] Pike KA (2014). Protein tyrosine phosphatase 1B is a regulator of the interleukin-10—Induced transcriptional program in macrophages. Sci. Signal..

[65] Chamberlain TC (2020). Interleukin-10 and small molecule SHIP1 allosteric regulators trigger anti-inflammatory effects through SHIP1/STAT3 complexes. iScience.

[66] Saqib U (2018). Phytochemicals as modulators of M1–M2 macrophages in inflammation. Oncotarget.

[67] Jiang J (2016). Macrophage polarization in IL-10 treatment of particle-induced inflammation and osteolysis. Am. J. Pathol..

[68] Shayganni E, Bahmani M, Asgary S, Rafieian-Kopaei M (2016). Inflammaging and cardiovascular disease: management by medicinal plants. Phytomedicine.

[69] Ferrucci L (2005). The origins of age-related proinflammatory state. Blood.

[70] Paolisso G (1998). Advancing age and insulin resistance: role of plasma tumor necrosis factor-α. Am. J. Physiol. Endocrinol. Metab..

[71] Koelman L, Pivovarova-Ramich O, Pfeiffer AFH, Grune T, Aleksandrova K (2019). Cytokines for evaluation of chronic inflammatory status in ageing research: reliability and phenotypic characterisation. Immun. Ageing.

[72] Minciullo PL (2016). Inflammaging and anti-inflammaging: the role of cytokines in extreme longevity. Arch. Immunol. Ther. Exp. (Warsz).

[73] Chung HY (2019). Redefining chronic inflammation in aging and age-related diseases: proposal of the senoinflammation concept. Aging Dis..

[74] Ferrucci L, Fabbri E (2018). Inflammageing: chronic inflammation in ageing, cardiovascular disease, and frailty. Nat. Rev. Cardiol..

[75] Teissier T, Boulanger É (2019). The receptor for advanced glycation end-products (RAGE) is an important pattern recognition receptor (PRR) for inflammaging. Biogerontology.

[76] Ishida T, Ishida M, Tashiro S, Takeishi Y (2019). DNA damage and senescence-associated inflammation in cardiovascular disease. Biol. Pharm. Bull..

[77] Covarrubias AJ (2020). Senescent cells promote tissue NAD+ decline during ageing via the activation of CD38+ macrophages. Nat. Metab..

[78] Zheng XF (2013). Lipopolysaccharide-induced M2 to M1 macrophage transformation for IL-12p70 production is blocked by candida albicans mediated up-regulation of EBI3 expression. PLoS ONE.

[79] Zhang Q, Lu Y, Bian H, Guo L, Zhu H (2017). Activation of the α7 nicotinic receptor promotes lipopolysaccharide-induced conversion of M1 microglia to M2. Am. J. Transl. Res..

[80] Karuppagounder V (2017). The senescence accelerated mouse prone 8 (SAMP8): a novel murine model for cardiac aging. Ageing Res. Rev..

[81] Griñan-Ferré C (2016). Environmental enrichment modified epigenetic mechanisms in SAMP8 mouse hippocampus by reducing oxidative stress and inflammaging and achieving neuroprotection. Front. Aging Neurosci..

[82] Sun J (2018). miR-330-5p/Tim-3 axis regulates macrophage M2 polarization and insulin resistance in diabetes mice. Mol. Immunol..

[83] Lu J (2013). Discrete functions of M2a and M2c macrophage subsets determine their relative efficacy in treating chronic kidney disease. Kidney Int..

[84] Liu C (2013). Targeting the shift from M1 to M2 macrophages in experimental autoimmune encephalomyelitis mice treated with Fasudil. PLoS ONE.

[85] *United States Pharmacopeia 41 Garlic Fluid Extract USP28-NF33* (United States Pharmacopeial Convention, 2018).

[86] Matsutomo T, Kodera Y (2016). Development of an analytic method for sulfur compounds in aged garlic extract with the use of a postcolumn high performance liquid chromatography method with sulfur-specific detection. J. Nutr..

[87] Amano H, Kazamori D, Itoh K (2016). Pharmacokinetics and N-acetylation metabolism of S-methyl-l-cysteine and trans-S-1-propenyl-l-cysteine in rats and dogs. Xenobiotica.

[88] Institute of Laboratory Animal Resources (US) (2011). Guide for the Care and Use of Laboratory Animals.

[89] Dunn KW, Kamocka MM, McDonald JH (2011). A practical guide to evaluating colocalization in biological microscopy. Am. J. Physiol. Cell Physiol..

