# Supplementary material for: S-1-Propenylcysteine promotes IL-10-induced M2c macrophage polarization through prolonged activation of IL-10R/STAT3 signaling
Source: Sci Rep. 2021 Nov 17;11:22469. doi: 10.1038/s41598-021-01866-3 (PMC8599840; doi:10.1038/s41598-021-01866-3)
Supplement: Supplementary file 1 — Supplementary Information. [file 41598_2021_1866_MOESM1_ESM.pdf]

Supplementary Information

S-1-Propenylcysteine promotes IL-10-induced M2c macrophage polarization through prolonged activation of IL-10R/STAT3 signaling

Satomi Miki[1, 2*], Jun-ichiro Suzuki[2], Miyuki Takashima[2],
Mari Ishida[1], Hiroki Kokubo[1] and Masao Yoshizumi[1*]

[1] Department of Cardiovascular Physiology and Medicine, Graduate School of Biomedical and Health Sciences, Hiroshima University, Hiroshima, 1-2-3 Kasumi, Minami-ku, Hiroshima-shi, Hiroshima, 734-8551, Japan.
[2] Central Research Institute, Wakunaga Pharmaceutical Co., Ltd., Hiroshima, 1624 Shimokotachi, Koda-cho, Akitakata-shi, Hiroshima, 739-1195, Japan.

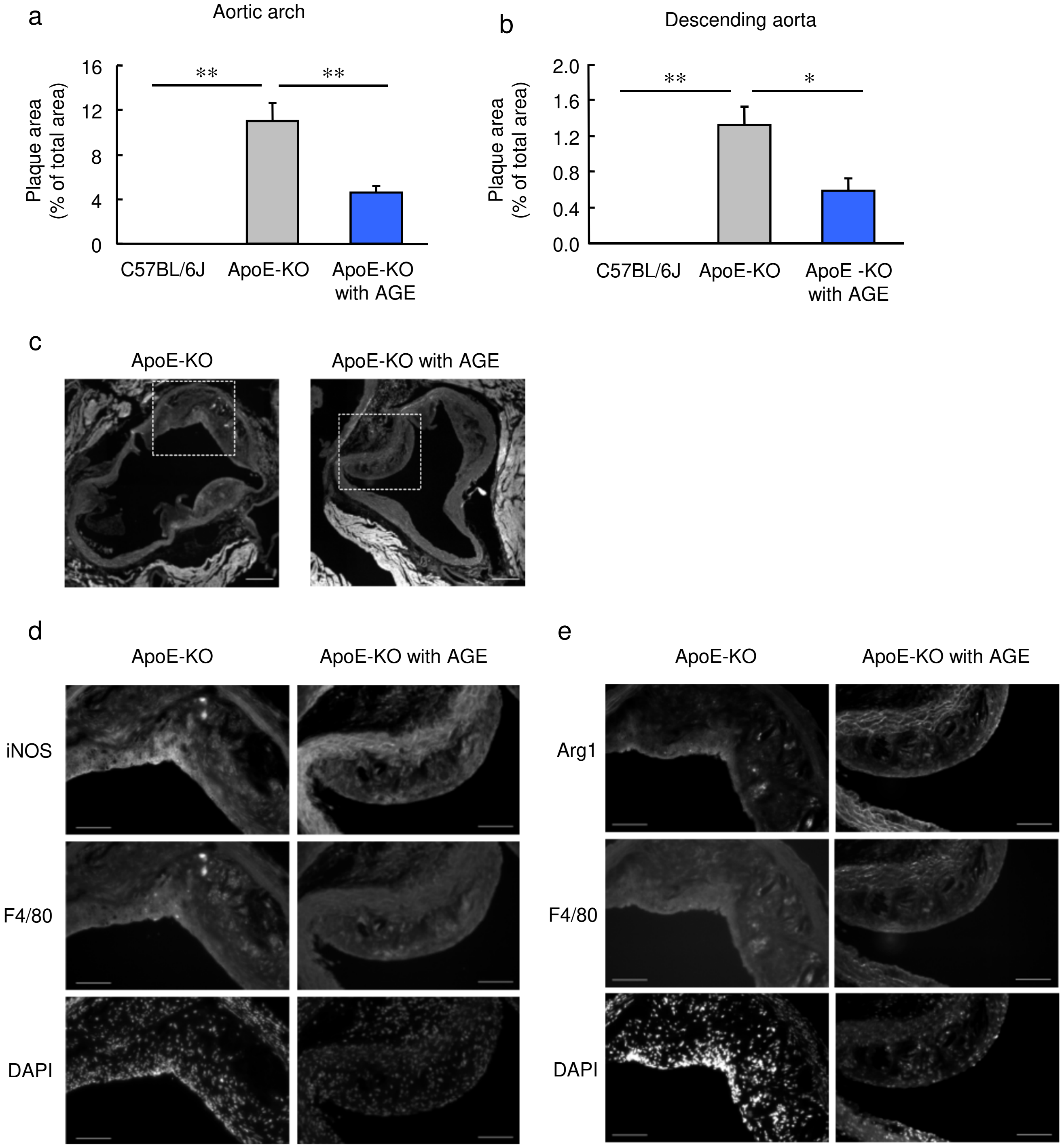

Supplemental Figure S1. Effect of AGE on lipid deposition in aortic arches and descending aortas of ApoE-KO mice. C57BL/6J (Control) and ApoE-KO mice were fed a diet containing 3% AGE for 17 weeks. (a, b) The percentages of Oil Red O-positive area in the (a) aortic arches and (b) descending aortas are shown in the bar graphs (n=5–8/group). (c) Slices of aortic roots were immunostained with anti-F-actin. Scale bar: 200 µm. (d, e) Enlargement of the white squares in (c). Aortas were stained with anti-iNOS, anti-Arg1, anti-F4/80, and DAPI. Scale bar: 100 µm. Data are shown as mean+SEM. Statistical differences were determined using Bonferroni's multiple comparison test (*p<0.05 and **p<0.01).

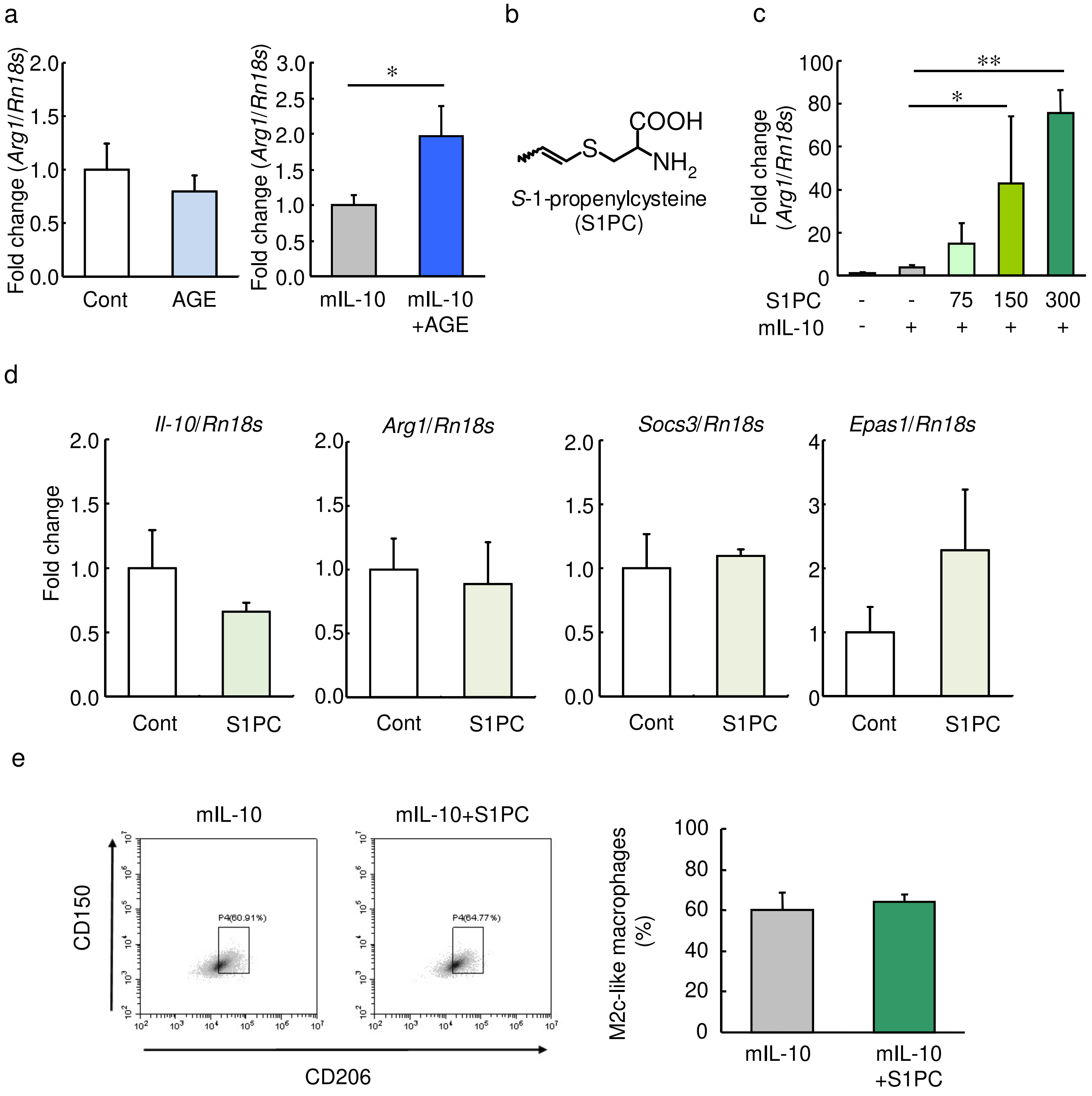

Supplemental Figure S2. Effect of AGE on mIL-10-induced *Arg1* mRNA level, and the effect of S1PC on mIL-10-induced macrophage polarization in BMDMs. (a) M-CSF-induced BMDMs were treated with AGE (2 mg/mL) in the presence or absence of mIL-10 (20 ng/mL) for 24 h. The relative level of *Arg1* mRNA were analyzed using qRT-PCR. (b) The chemical structure of S1PC. (c) M-CSF-induced BMDMs were treated with S1PC (75–300 μM) in the presence of mIL-10 (20 ng/mL) for 48 h. The relative level of *Arg1* mRNA were analyzed using qRT-PCR. (d) M-CSF-induced BMDMs were treated with S1PC (300 μM) for 48 h. The relative levels of *Il-10*, *Arg1*, *Socs3,* and *Epas1* mRNA were analyzed using qRT-PCR. (e) M-CSF-induced BMDMs were treated with S1PC (300 μM) in the presence of mIL-10 (20 ng/mL) for 24 h. The population of M2c-like macrophages ($CD11b^+$, $F4/80^+$, $CD86^-$, $CD206^+$, and $CD150^+$ cells) were analyzed by flow cytometry. Data are shown as mean+SD. Data are representative of three independent experiments. Statistical differences were determined using (a) Student's *t*-test or (c) Bonferroni's multiple comparison test (*$p<0.05$ and **$p<0.01$).

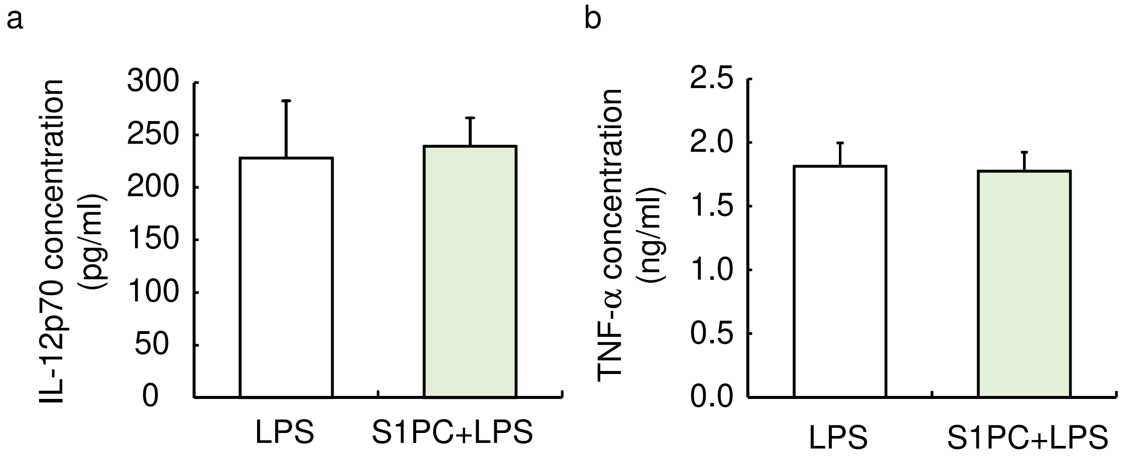

Supplemental Figure S3. Effect of S1PC on LPS-induced production of inflammatory cytokines in BMDMs. GM-CSF-induced BMDMs were treated with S1PC (300 μM) for 48 h. Then, they were washed with PBS and treated with LPS (100 ng/mL) for 16 h. The amounts of (a) IL-12p70 and (b) TNF-α secreted into the culture media were determined using ELISA. Data are shown as mean+SD. Data are representative of three independent experiments.

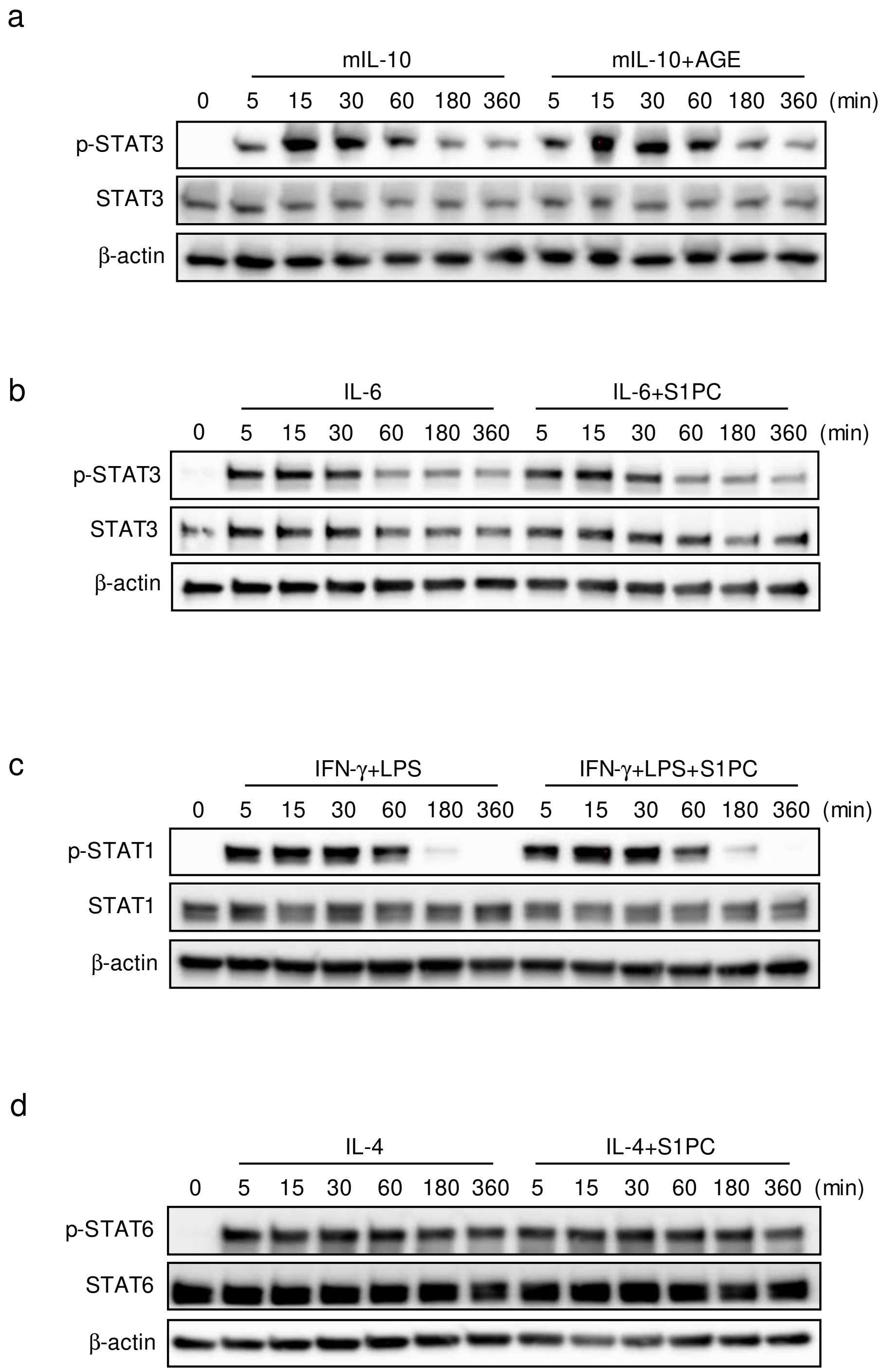

Supplemental Figure S4. Effect of AGE on mIL-10-induced STAT3 phosphorylation level and effect of S1PC on IL-6, IFN-γ plus LPS, and IL-4-induced STAT signaling pathway in BMDMs. (a) M-CSF-induced BMDMs were treated with AGE (2 mg/mL) in the presence of mIL-10 (2 ng/mL) for 5 to 360 min. The cell lysates were analyzed using immunoblotting with the indicated antibodies. The full-length blots are shown in Supplemental Figure S17a. (b–d) M-CSF-induced BMDMs were treated with S1PC (300 μM) in the presence of (b) IL-6 (10 ng/mL), (c) IFN-γ (5 ng/mL) plus LPS (25 ng/mL), and (d) IL-4 (5 ng/mL) for 5 to 360 min. The cell lysates were analyzed by immunoblotting with the indicated antibodies. The full-length blots are shown in Supplemental Figure S17b–d.

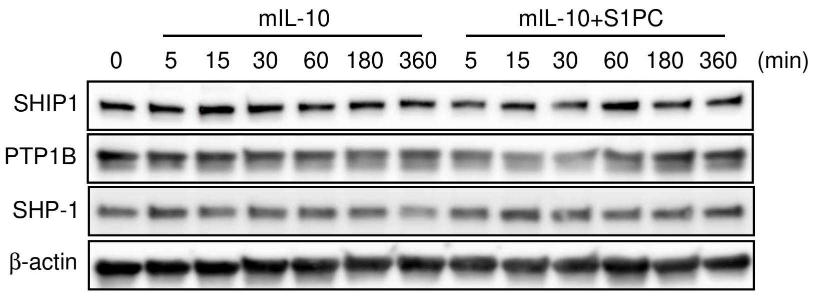

Supplemental Figure S5. Effect of S1PC on the protein levels of protein phosphatases. M-CSF-induced BMDMs were treated with S1PC (300 μM) in the presence of mIL-10 (2 ng/mL) for 5 to 360 min. The cell lysates were analyzed using immunoblotting with the indicated antibodies. The full-length blots are shown in Supplemental Figure S18.

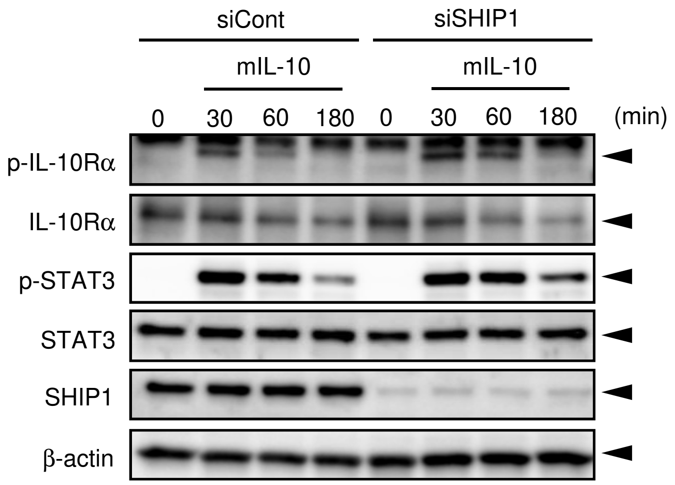

Supplemental Figure S6. Effect of SHIP1 knockdown on the IL-10 signaling pathway. J774A.1 cells were transfected with control siRNA (siControl) or Inpp5d (SHIP1) siRNA (siSHIP1). siRNA transfected cells were treated with mIL-10 (10 ng/mL) for 30 to 180 min. The cell lysates were analyzed using immunoblotting with the indicated antibodies. The full-length blots are shown in Supplemental Figure S19a.

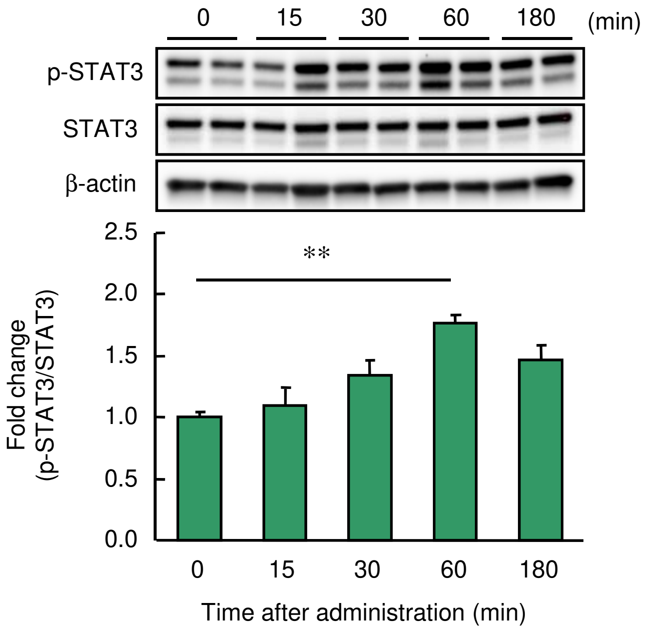

Supplemental Figure S7. Effect of S1PC on STAT3 phosphorylation in SAMP8 mice. The SAMP8 mice were single orally administered S1PC (5 mg/kg), and their spleens was removed at 0 to 180 min after administration. The relative protein level of phospho-STAT3 in the splenic lymphocytes were analyzed using immunoblotting with the indicated antibodies (n=5/group). The full-length blots are shown in Supplemental Figure S19b. Data are shown as mean+SEM. Statistical differences were determined using Bonferroni's multiple comparison test (**p<0.01).

	TG (mg/ml)	TC (mg/ml)	HDL-C (mg/ml)	NEFA (μEq/ml)
C57BL/6J	0.90 ± 0.08	0.55 ± 0.10	1.02 ± 0.07	0.66 ± 0.04
ApoE-KO	1.92 ± 0.24 **	6.32 ± 0.71 **	0.38 ± 0.07 **	0.93 ± 0.08 **
ApoE-KO with AGE	1.82 ± 0.15	5.80 ± 0.49	0.38 ± 0.06	0.90 ± 0.08

Supplemental Table S1. Effect of AGE on the systemic lipid profiles of ApoE-KO mice. C57BL/6J (Control) and ApoE-KO mice were fed a diet containing 3% AGE for 17 weeks (n=5-8/group). Plasma levels of TG, TC, HDL-C, and NEFA were quantified using commercially available kits (See methods). Data are shown as mean±SEM. Statistical differences were determined using Bonferroni's multiple comparison test (**$p<0.01$).

Antibodies	Maker (Cat.No)
For immunoblotting	
Anti-phospho-Jak1 (Tyr^{1034}/Tyr^{1035})	Cell Signaling Technology (#74129)
Anti-Jak1	Cell Signaling Technology (#50996)
Anti-phospho-Stat3 (Tyr^{705})	Cell Signaling Technology (#9145)
Anti-Stat3	Cell Signaling Technology (#9139)
Anti-phospho-Stat1 (Tyr^{701})	Cell Signaling Technology (#7649)
Anti-Stat1	Cell Signaling Technology (#14994)
Anti-phospho-Stat6 (Tyr^{641})	Cell Signaling Technology (#56554)
Anti-SHP-1	Cell Signaling Technology (#3759)
Anti-arginase-1	Proteintech (#16001-1-AP)
Anti-IL-10	Proteintech (#20850-1-AP)
Anti-CD206	Proteintech (#18704-1-AP)
Anti-Stat6	Proteintech (#66717-1-Ig)
Anti-SHIP1	Proteintech (#19694-1-AP)
Anti-PTP1B	Proteintech (#11334-1-AP)
Anti-phospho-IL-10Rα (Tyr^{496})	Bioworld technology (#BS4592)
Anti-IL-10Rα	R&D Systems (#AF-474-NA)
Anti-iNOS	abcam (#ab15323)
Anti-β-actin	FUJIFILM Wako Pure Chemical Corporation (#010-27841)
Anti-β-actin	MEDICAL & BIOLOGICAL LABORATORIES (PM053-7)
HRP-conjugated Rabbit IgG	Cell Signaling Technology (#7074)
HRP-conjugated Mouse IgG	Cell Signaling Technology (#7076)
HRP-conjugated Rat IgG	Cell Signaling Technology (#7077)
HRP-conjugated Goat IgG	Promega (#V8051)
For immunoprecipitation	
Anti-IL-10Rα	Santa cruz biotechnology (#sc-365374)
Anti-SHIP1	Proteintech (#19694-1-AP)
For flow cytometry	
Anti-CD11b-APC-Vio770	Miltenyi Biotec (#130-109-288)
Anti-CD86-APC	Miltenyi Biotec (#130-102-558)
Anti-F4/80-AlexaFluor488	Thermo Fisher Scientific (#53-4801-82)
Anti-CD206-eFluor450	Thermo Fisher Scientific (#48-2061-80)
Anti-CD150-PE	BD Biosciences (#562651)
For Immunohistological staining	
Anti-iNOS	abcam (#ab15323)
Anti-arginase-1	Cell Signaling Technology (#93668)
Anti-F4/80-AlexaFluor647	Biolegend (#123122)
Anti-rabbit IgG-AlexaFluor488	Cell Signaling Technology (#4412)
For immunofluorescence staining	
Anti-Stat3	Proteintech (#60199-1-Ig)
Anti-Mouse IgG-AlexaFluor594	Thermo Fisher Scientific (#A-11005)

Supplemental Table S2. Antibodies used in this study.

Genes		Primer sequences
mouse *Rn18s*	forward	5'-ATGCGGCGGCGTTATTCC-3'
mouse *Rn18s*	reverse	5'-ATCTGTCAATCCTGTCCGTGTC-3'
mouse *Cd68*	forward	5'-CACGCGTCCGGGAACTGG-3'
mouse *Cd68*	reverse	5'-GGATGGCAGGAGAGTAACGGC-3'
mouse *Hif1α*	forward	5'-GGACAGAGCCGGCGTTTAGG-3'
mouse *Hif1α*	reverse	5'-TTCTTCTCGTTCTCGCCGCC-3'
mouse *Ccl5*	forward	5'-CCAGCAGCAAGTGCTCCAAT-3'
mouse *Ccl5*	reverse	5'-TTCTCTGGGTTGGCACACACT-3'
mouse *Arg1*	forward	5'-TGGCTTGCGAGACGTAGACC-3'
mouse *Arg1*	reverse	5'-ATCACCTTGCCAATCCCCAGC-3'
mouse *Mrc1* (CD206)	forward	5'-AGGGACGTTTCGGTGGACTG-3'
mouse *Mrc1* (CD206)	reverse	5'-ACCTGCCACTCCGGTTTTCA-3'
mouse *Epas1* (HIF2α)	forward	5'-AAAGACGTGTCCACCGAGCG-3'
mouse *Epas1* (HIF2α)	reverse	5'-GTGCAGTGCAGGACGGACTT-3'
mouse *Msr1* (SR-AI)	forward	5'-ACCTGTGCCCAGCCTGTTTC-3'
mouse *Msr1* (SR-AI)	reverse	5'-CCTGTGCCCAAGGTGAGGTG-3'
mouse *Slamf1* (CD150)	forward	5'-ACTTGGTGAGCGTGGAGGAG-3'
mouse *Slamf1* (CD150)	reverse	5'-GCAAGCTGCAGGTCCCATTC-3'
mouse *Il-10*	forward	5'-TGCTCCTAGAGCTGCGGACT-3'
mouse *Il-10*	reverse	5'-GGCTTGGCAACCCAAGTAACC-3'
mouse *Socs3*	forward	5'-GCTGGTACTGAGCCGACCTC-3'
mouse *Socs3*	reverse	5'-GAGTCCAGGTGGCCGTTGAC-3'
mouse *Tnfα* Taqman	Mm00443258_m1 (Applied Biosystems)	
mouse *Il-18* Taqman	Mm00434226_m1 (Applied Biosystems)	

Supplemental Table S3. Primer sequences used in this study.

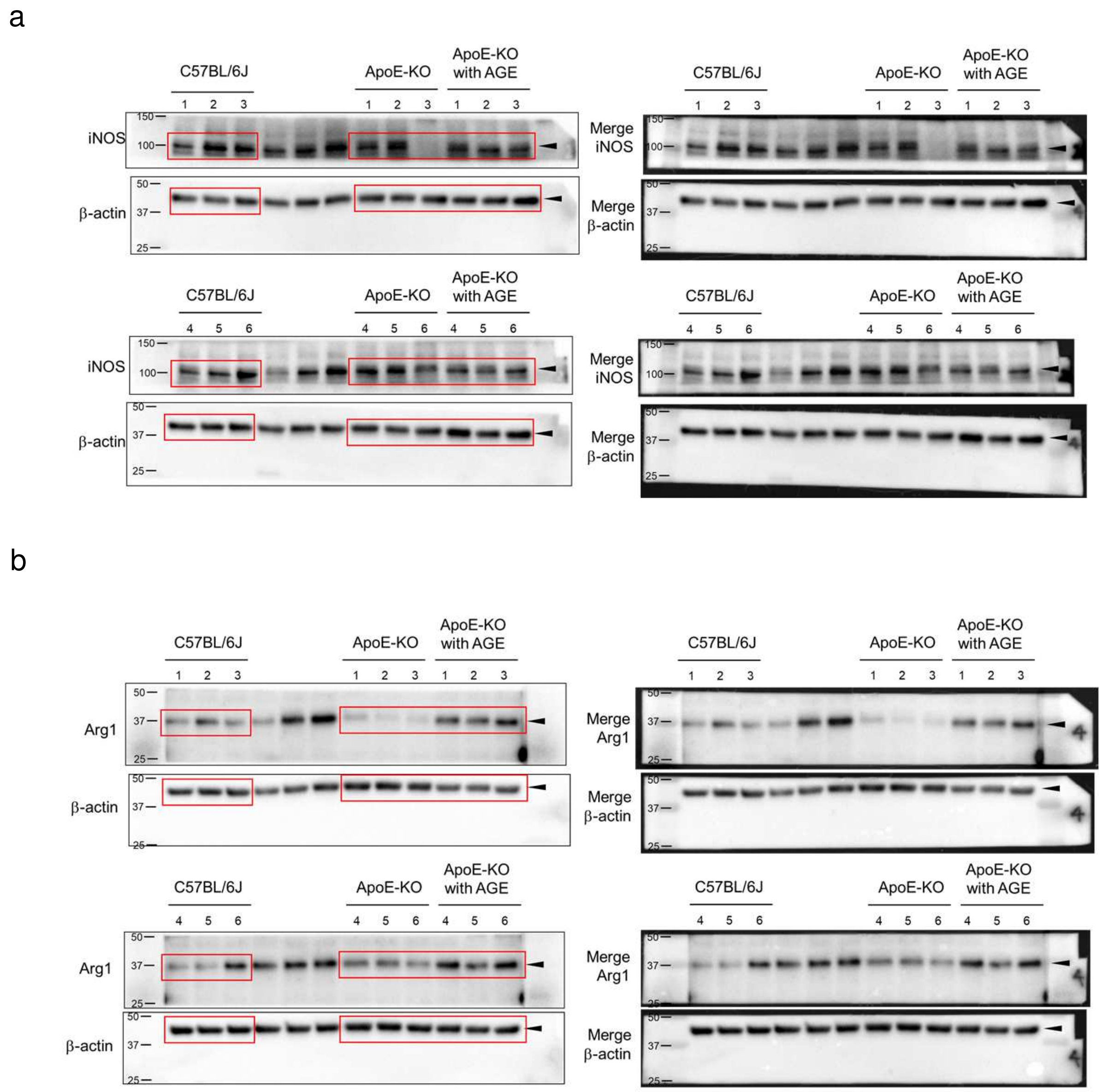

Supplemental Figure S8. Full-width membrane with a protein marker besides the original blots used in Figure 1d. The molecular size is shown as indicated. (a) and (b) were evaluated using the same samples in different membranes. The bands in the red lined squares were used for quantification.

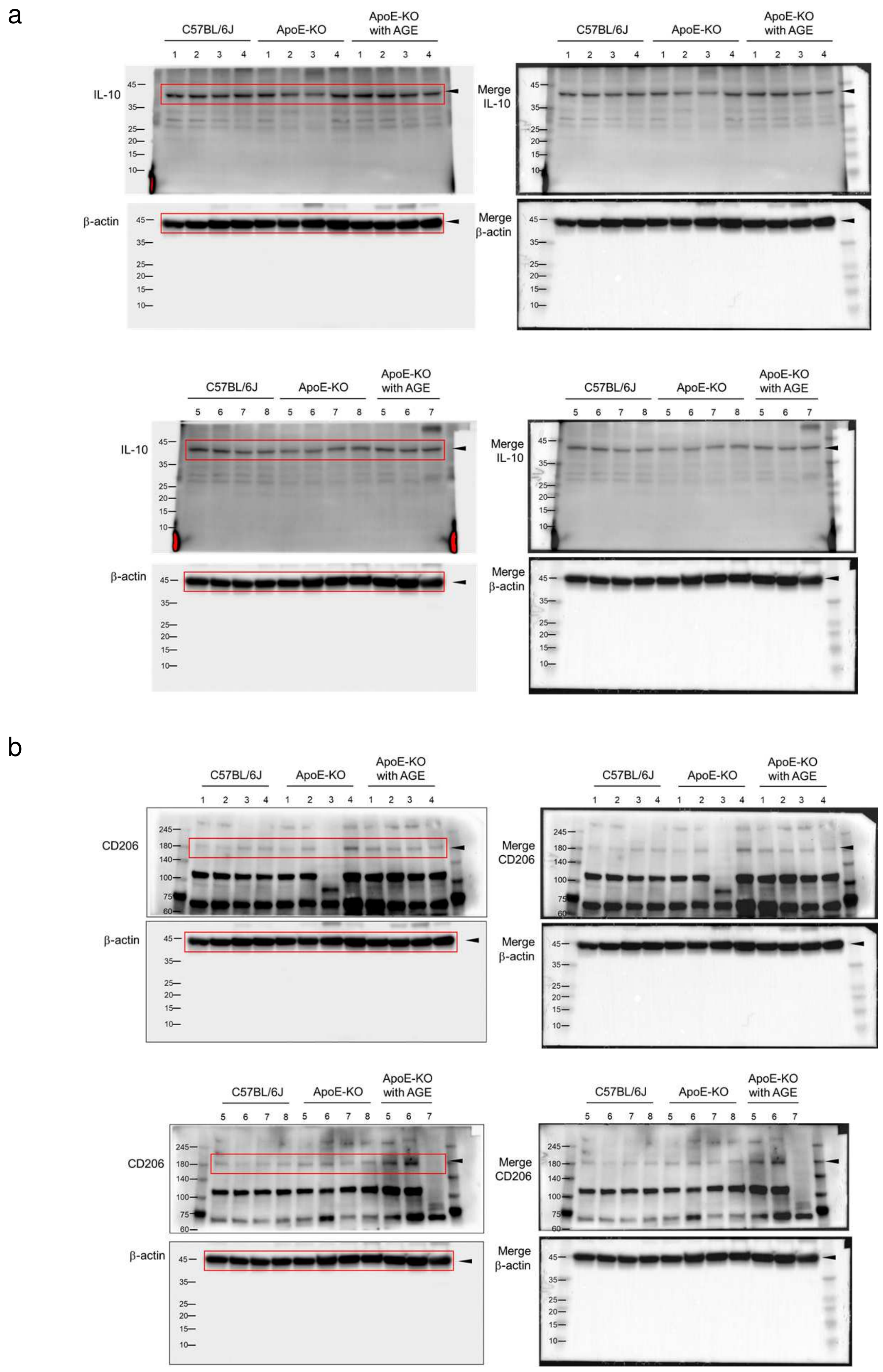

Supplemental Figure S9. Full-width membrane with a protein marker besides the original blots used in Figure 1d. The molecular size is shown as indicated. (a) and (b) were evaluated using the same samples in same membranes. The bands in the red lined squares were used for quantification.

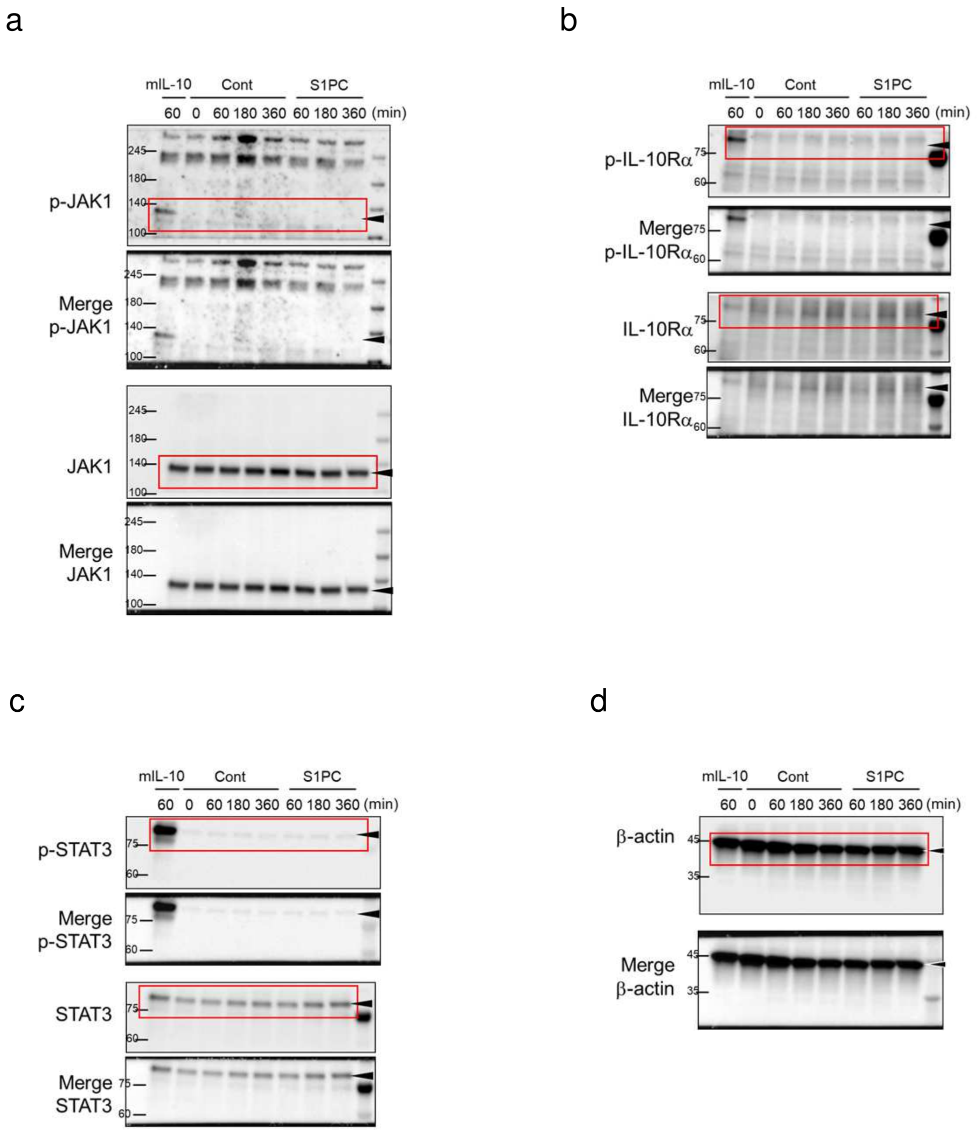

Supplemental Figure S10. Full-width membrane with a protein marker besides the original blots used in Figure 4a. The molecular size is shown as indicated. (a–d) All blots were evaluated using the same samples in same membrane. The bands in the red lined squares were used.

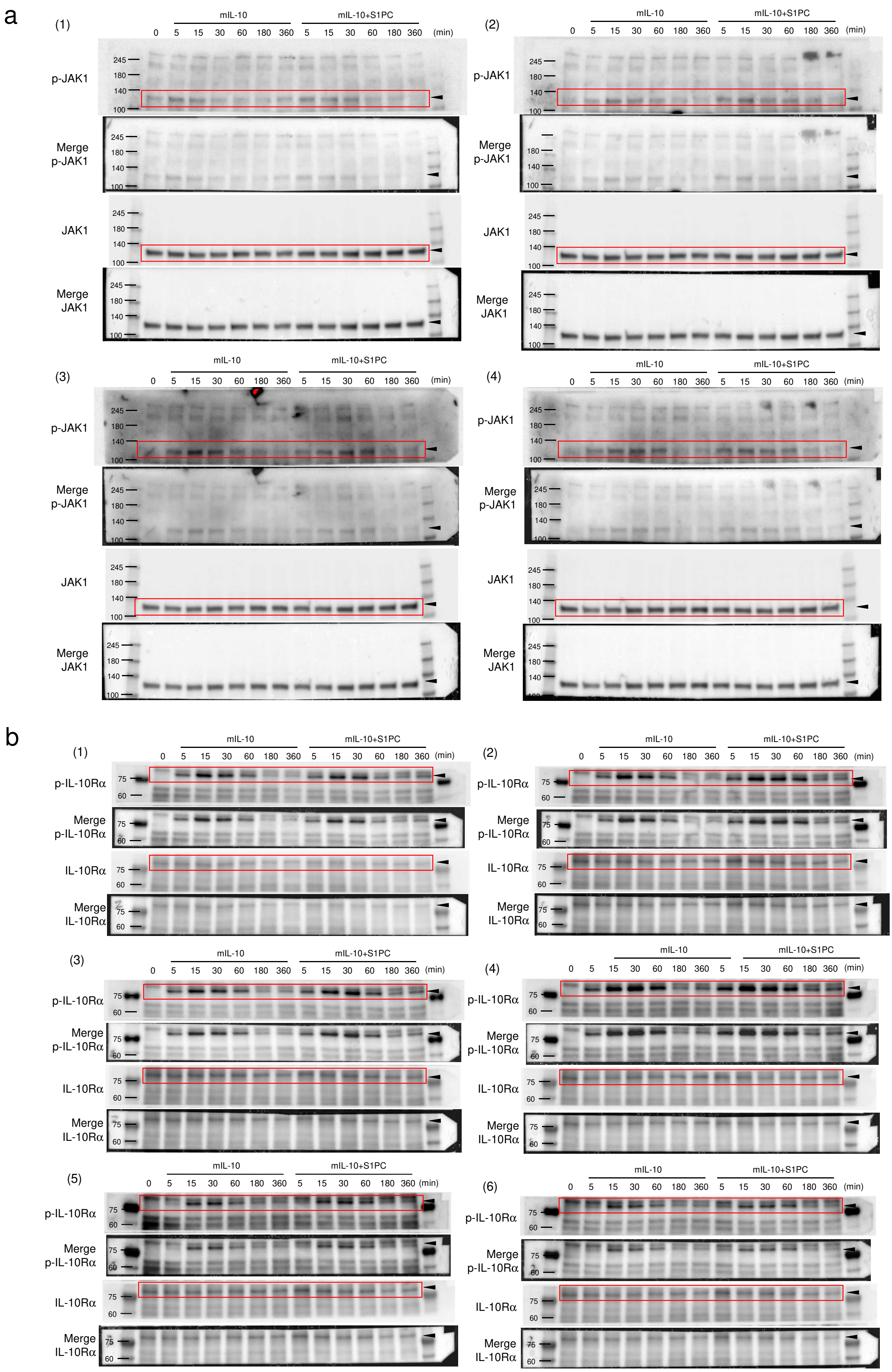

Supplemental Figure S11. Full-width membrane with a protein marker besides the original blots used in Figure 4b and 4e (a), and 4b and 4c (b). The molecular size is shown as indicated. (a) The quantification of p-JAK1 was performed in 4 independent experiments (1–4). (b) p-IL-10Rα was quantified in 6 independent experiments (1–6). a(1–4) and b(1–4) are cropped from the same membrane, respectively. For total JAK1 blotting, each membrane was stripped and re-blotted. For total IL-10Rα blotting, each same sample was newly separated using SDS-PAGE and blotted. The bands in the red lined squares were used for quantification.

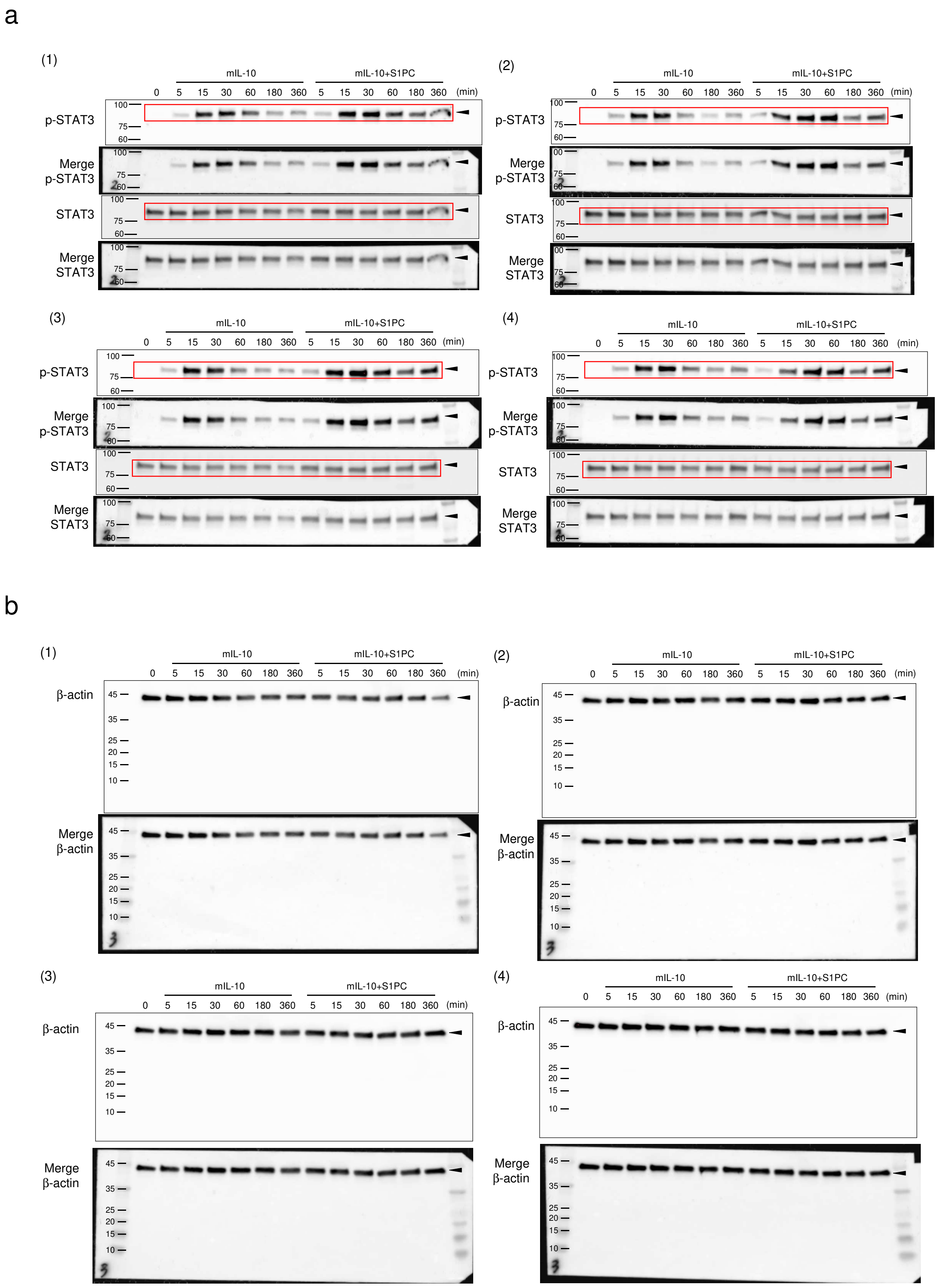

Supplemental Figure S12 Full-width membrane with a protein marker besides the original blots used in Figure 4b and 4d (a), and 4b (b). (a) The quantification of p-STAT3 was performed in 4 independent experiments (1–4). a(1–4) and b(1–4) are cropped from the same membrane, respectively. The bands in the red lined squares were used for quantification.

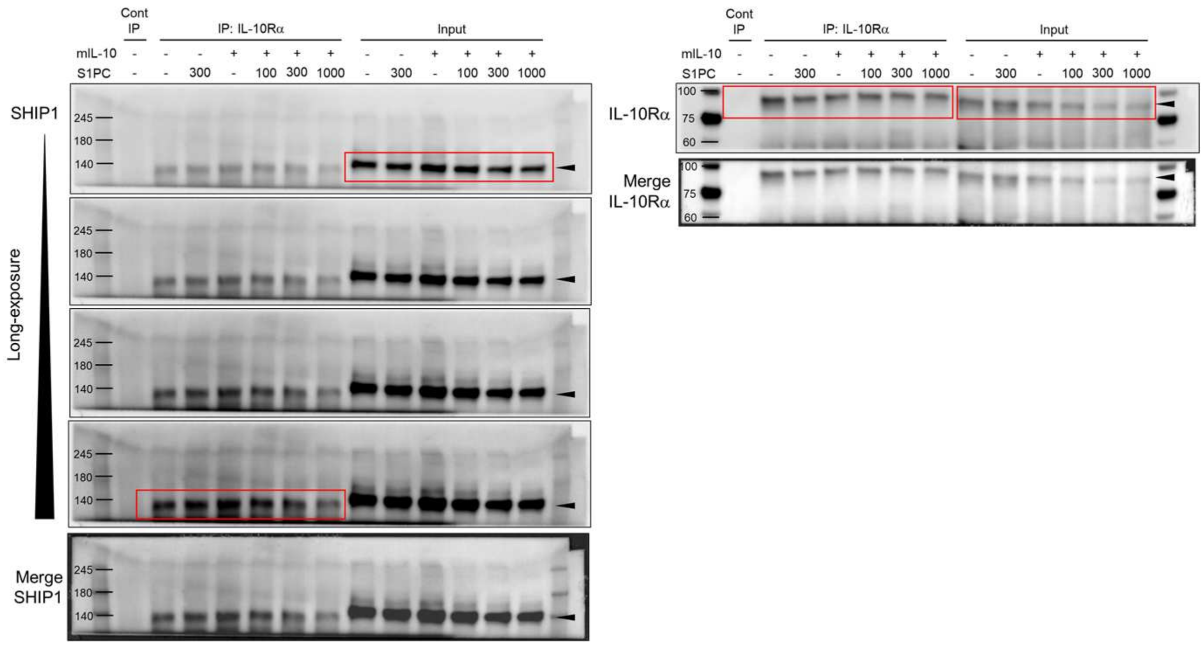

Supplemental Figure S13. Full-width membrane with a protein marker besides the original blots used in Figure 4g. The molecular size is shown as indicated. All blots were evaluated on the same membranes. The blots of SHIP1 were detected at several exposure times. The bands in the red lined squares were used.

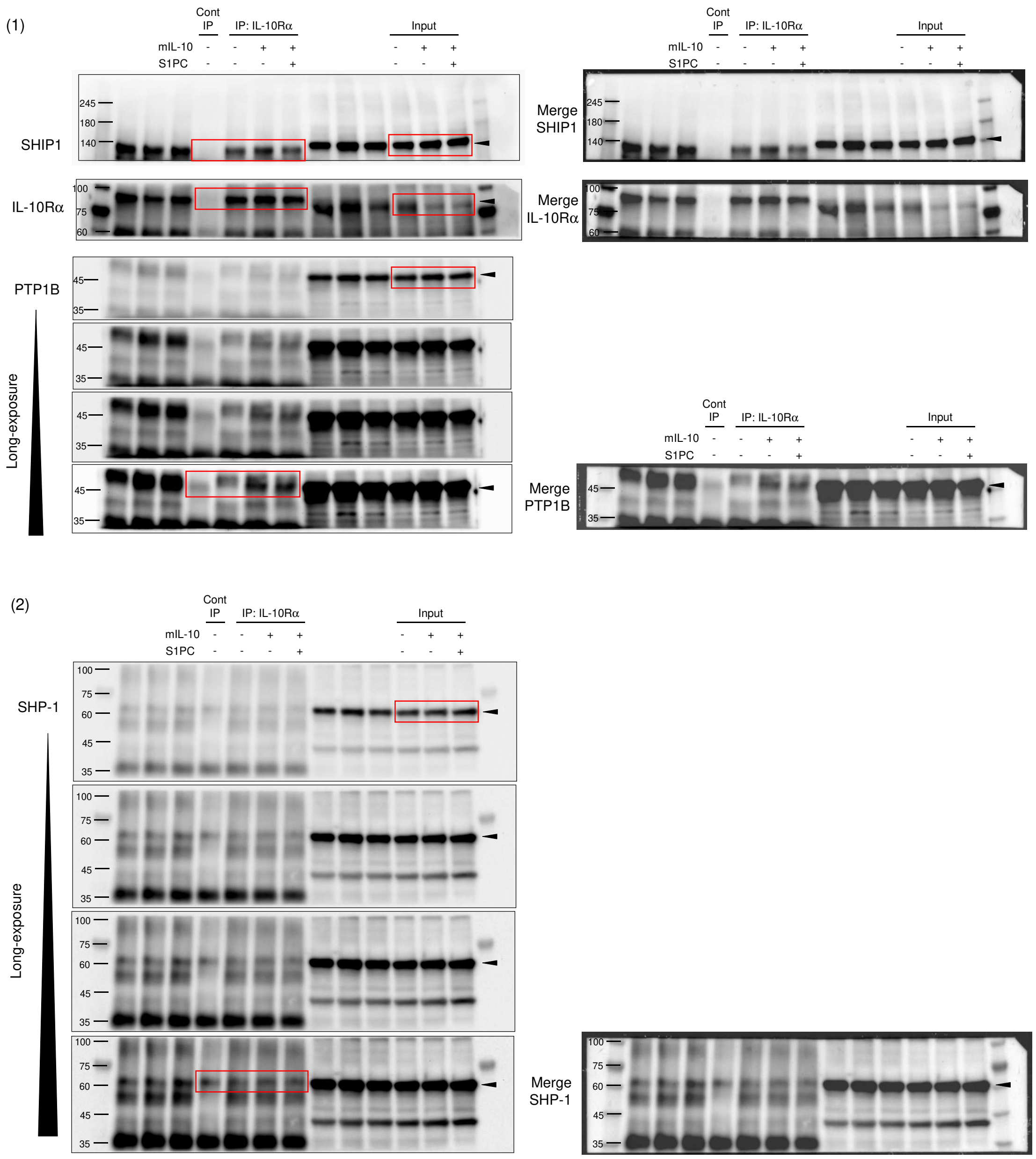

Supplemental Figure S14 Full-width membrane with a protein marker besides the original blots used in Figure 4h. The molecular size is shown as indicated. (1) and (2) are evaluated using the same samples in different membranes. The blots of PTP1B and SHP-1 were detected at several exposure times. The bands in the red lined squares were used.

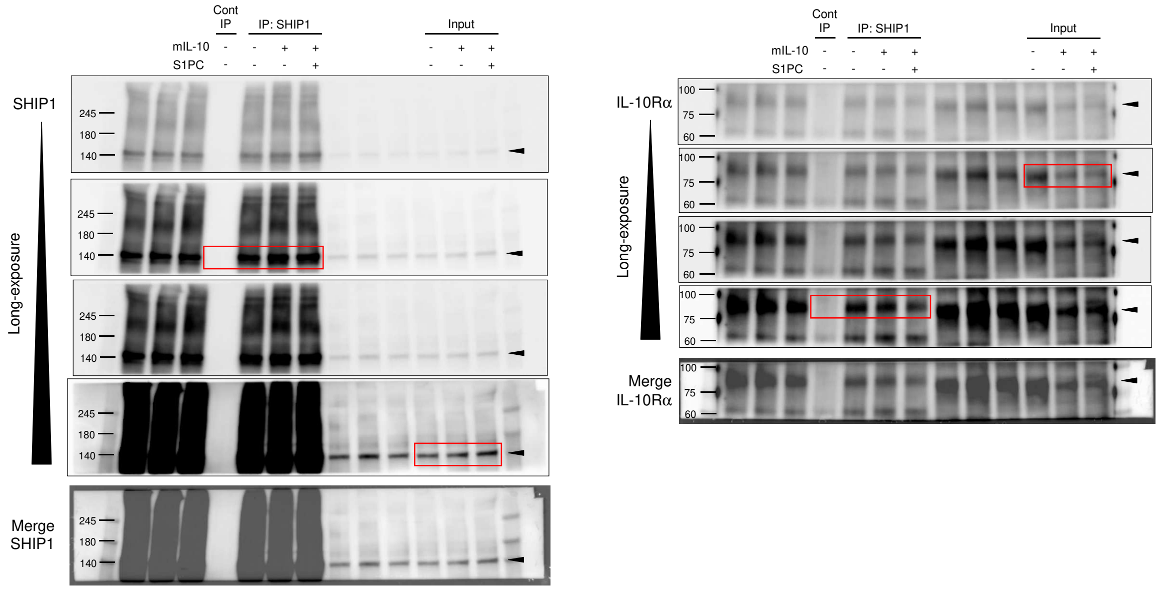

Supplemental Figure S15 Full-width membrane with a protein marker besides the original blots used in Figure 4i. The molecular size is shown as indicated. All blots were evaluated on the same membranes. The blots of SHIP1 and IL-10Rα were detected at several exposure times. The bands in the red lined squares were used.

a

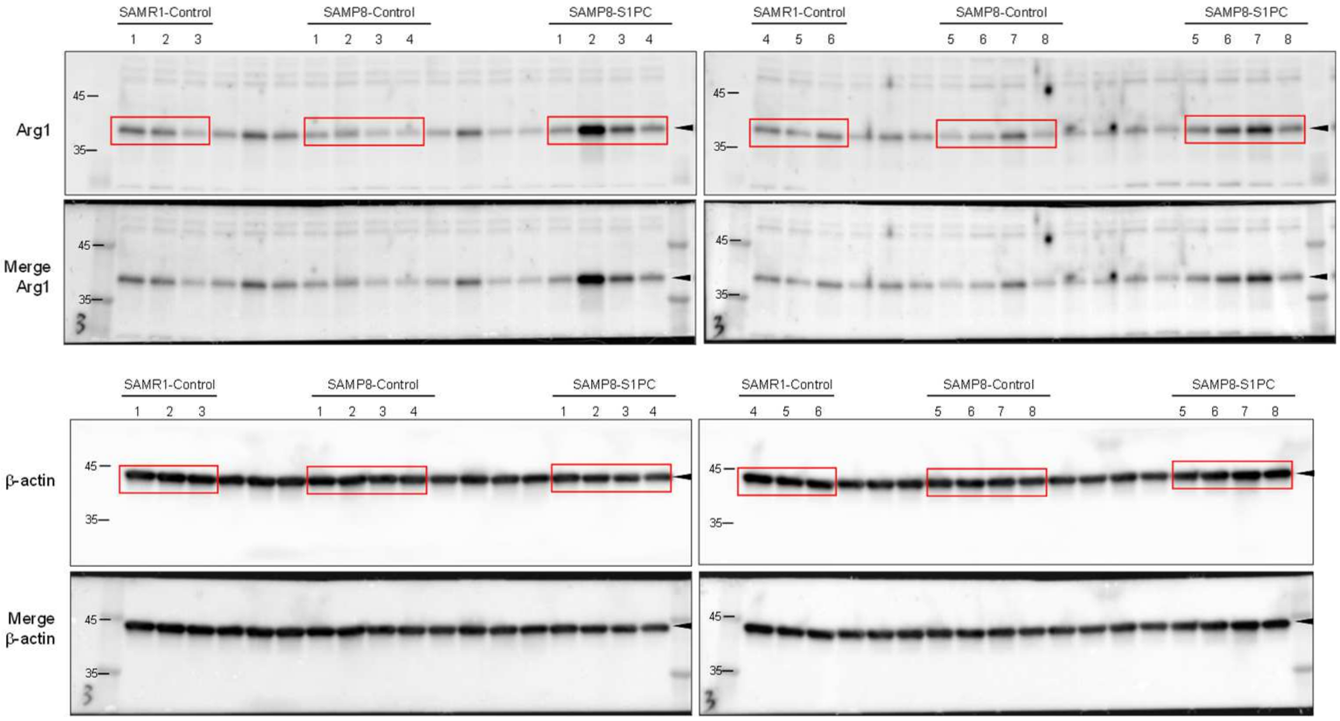

b

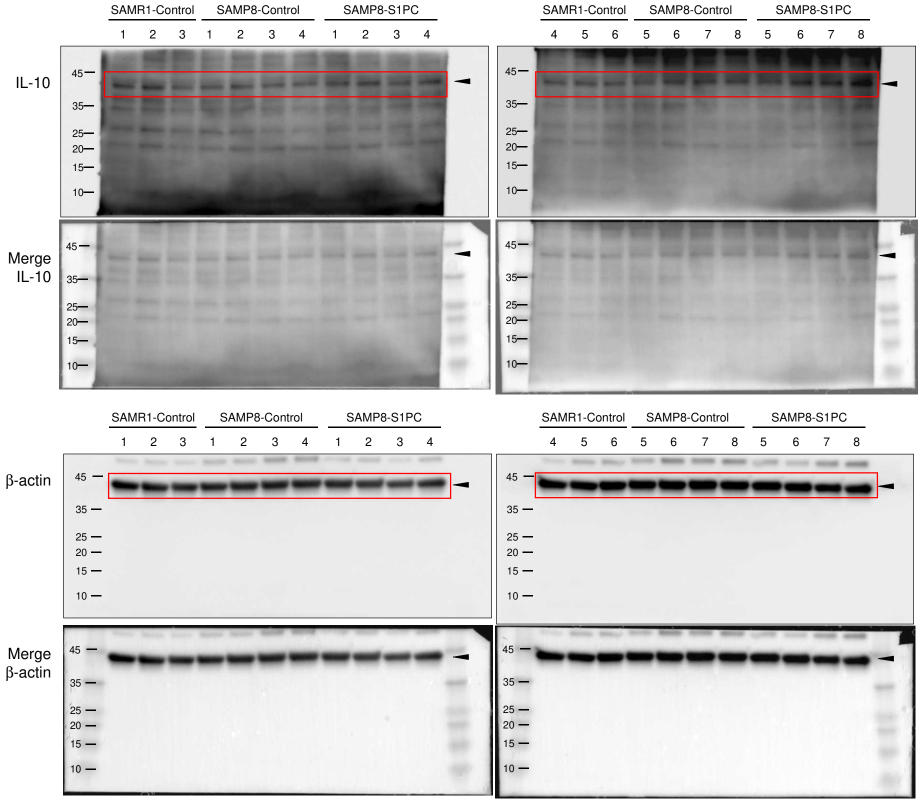

Supplemental Figure S16. Full-width membrane with a protein marker besides the original blots used in Figure 5b. The molecular size is shown as indicated. (a) and (b) were evaluated using the same samples in different membranes. The bands in the red lined squares were used for quantification.

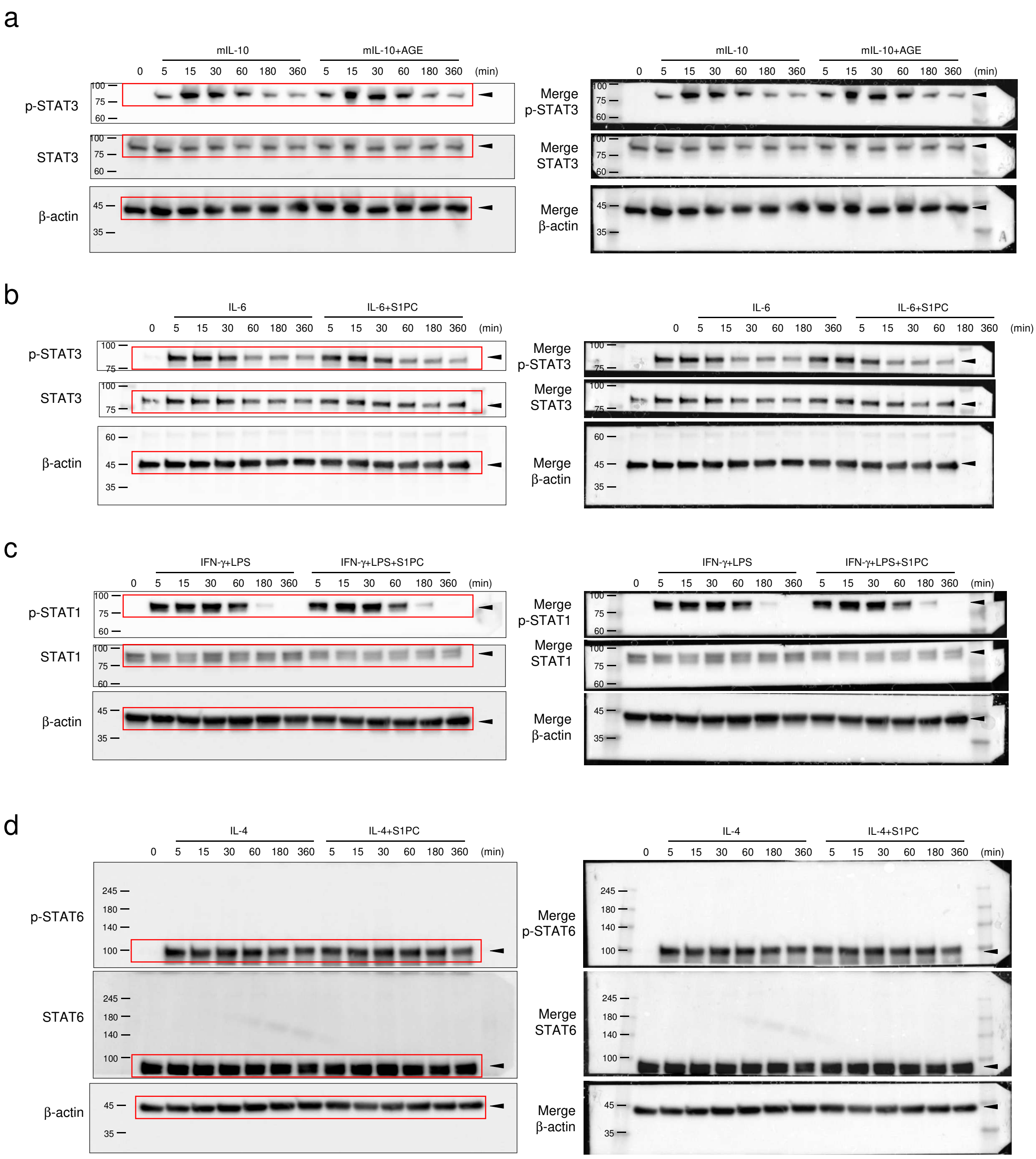

Supplemental Figure S17. Full-width membrane with a protein marker besides the original blots used in Supplemental Figure S4a (a), S4b (b), S4c (c), and S4d (d). The molecular size is shown as indicated. Protein expressions were evaluated using the same membrane in each figure of (a–d), respectively. The bands in the red lined squares were used.

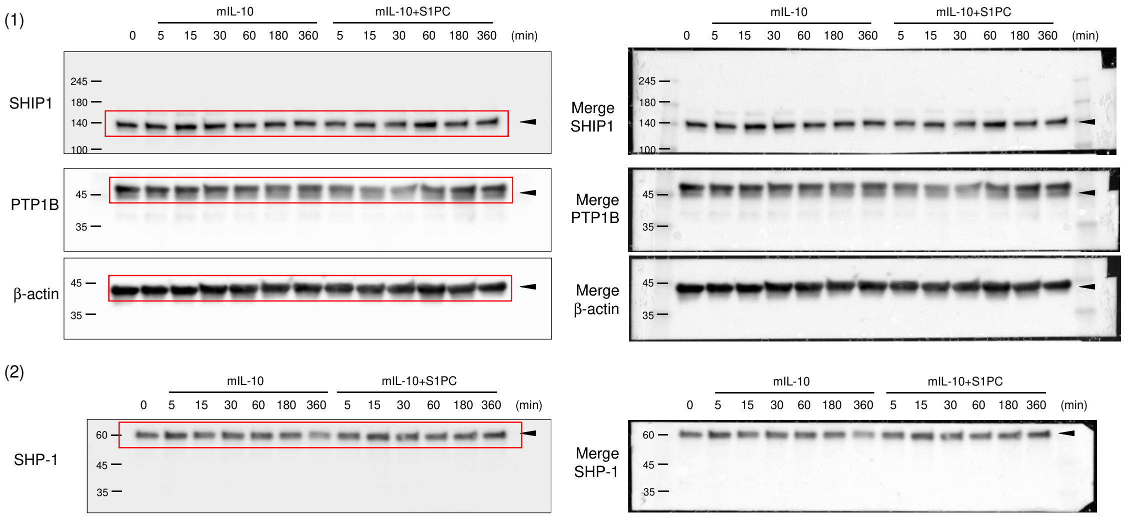

Supplemental Figure S18. Full-width membrane with a protein marker besides the original blots used in Supplemental Figure S5. The molecular size is shown as indicated. The blots of SHIP1, PTP1B, and β-actin were evaluated on the same membranes, while SHP-1 was evaluated on the another membrane. The bands in the red lined squares were used.

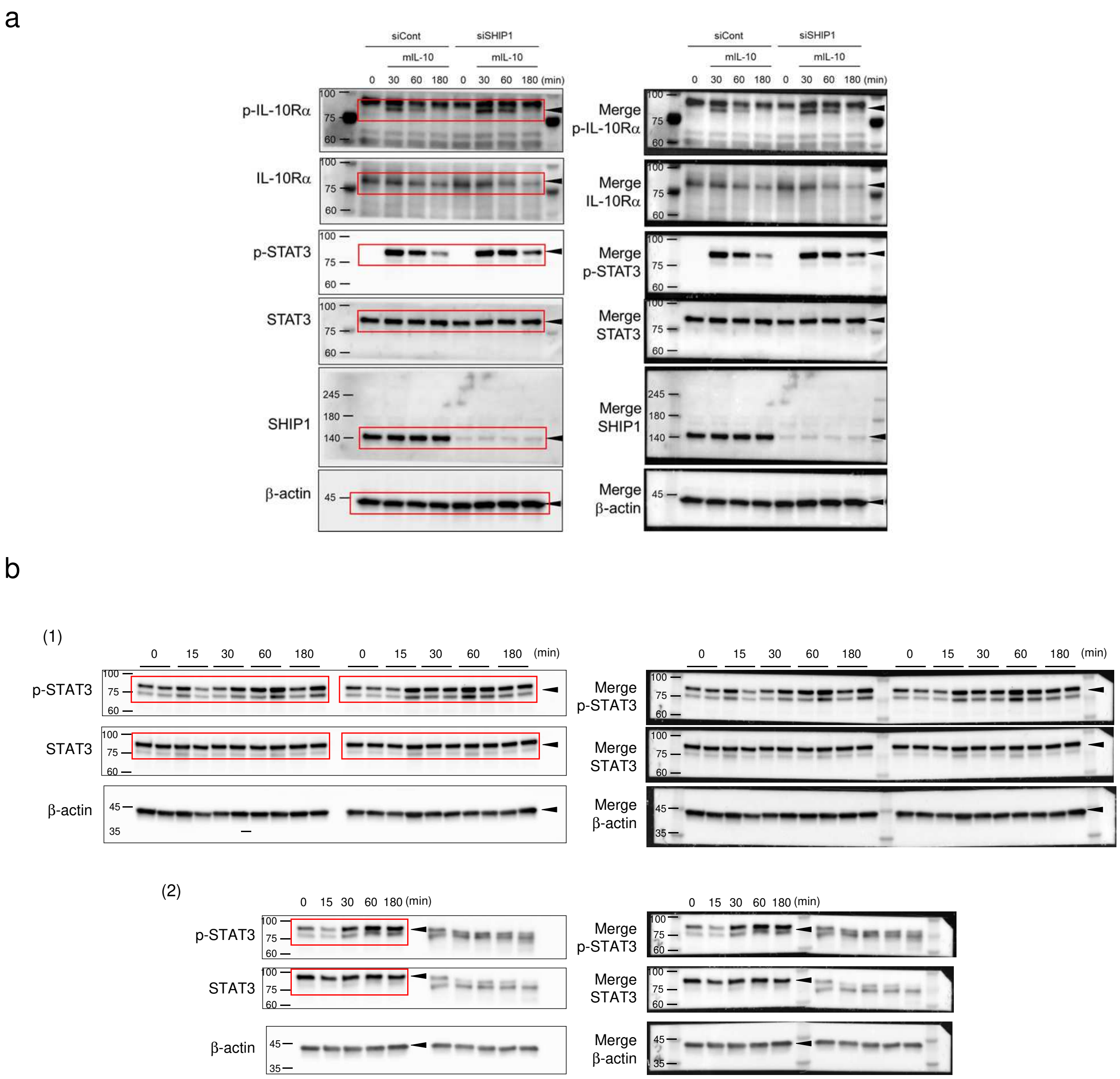

Supplemental Figure S19. Full-width membrane with a protein marker besides the original blots used in Supplemental Figure S6 (a) and S7 (b). The molecular size is shown as indicated. (a) All blots are evaluated on the same membranes, respectively. The bands in the red lined squares were used. (b) The blots for p-STAT3 were evaluated on two membranes (1–2). The bands in the red lined squares were used for quantification (n=5/group). .
